# Anode-Less (Anode-Free) Batteries: From Fundamental Principles to Practical Pathways Toward Solid-State Implementation

**DOI:** 10.3390/ma19061232

**Published:** 2026-03-20

**Authors:** Manuela Carvalho Baptista, Maria Helena Braga

**Affiliations:** 1 Faculty of Engineering, University of Porto, Rua Dr. Roberto Frias, 4200-465 Porto, Portugal; up200501276@edu.fe.up.pt; 2 MatER—Materials for Energy Research Laboratory, Faculty of Engineering, University of Porto, Rua Dr. Roberto Frias, 4200-465 Porto, Portugal; 3 LAETA, Institute of Science and Innovation in Mechanical and Industrial Engineering, Rua Dr. Roberto Frias, 4200-465 Porto, Portugal

**Keywords:** anode-less, anode-free, all-solid-state batteries, interface engineering, conditioning, characterization techniques, next-generation energy storage

## Abstract

**Highlights:**

**What are the main findings?**

- A comprehensive review on anode-less battery design for metal-ion systems.
- Detailed analysis of electrolyte design (liquid and solid) and interface engineering.
- Critical assessment of advanced *operando* characterization techniques for anode-less cells.
- Identifies plating/stripping efficiency and dendrite growth as key challenges.
- Solid-state electrolytes offer a promising path for safer, high-energy anode-less cells.

**What are the implications of the main findings?**

- Provides a roadmap for developing next-generation, high-energy-density batteries.
- Highlights the critical importance of current collector and interlayer functionalization.
- Underlines the necessity for standardized performance evaluation protocols.
- Bridges fundamental material insights with industrial scale-up requirements.
- Positions the anode-less architecture as a viable strategy for sustainable batteries.

**Abstract:**

Anode-less battery architectures, which eliminate the host anode material, have attracted considerable attention as a promising approach to increase energy density, simplify cell manufacturing, and improve safety in next-generation energy storage systems. This review provides a structured and integrative overview on the current research landscape of anode-less cells, spanning both liquid- and solid-electrolyte technologies. It first introduces the fundamental principles, key advantages, and inherent challenges of the anode-less concept. Advanced characterization techniques, including electrochemical, interfacial, morphological, and *operando* approaches, are then discussed as essential tools for probing metal plating/stripping behavior and degradation mechanisms. The core of the review examines how system design governs performance, addressing strategies for liquid electrolytes, including current collector design, electrolyte formulation, and deposition control, as well as solid electrolytes, with an emphasis on interfacial engineering, fundamental limitations, and extensions to Na- and K-based batteries. By integrating insights across these systems, the review identifies critical challenges, including unstable solid-electrolyte interphases, dendrite formation, and interfacial contact loss. Finally, a development pyramid is introduced as a conceptual framework linking fundamental research to practical implementation, outlining key priorities from interface control and full-cell compatibility to long-term reliability while also highlighting industrial pathways toward hybrid and fully solid-state anode-less batteries.

## 1. Introduction

**Figure 1:**
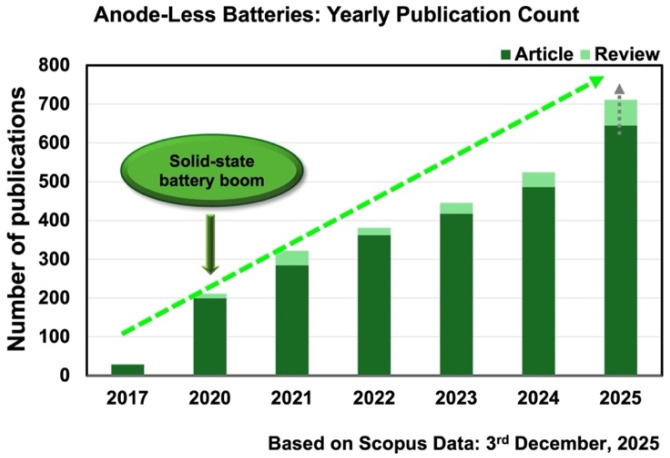
Annual evolution of the number of publications related to anode-less batteries between 2017 and 2025, distinguishing research articles from review articles. Data obtained from the Scopus database on 3 December 2025.

Anode-less battery configurations eliminate the need for host anode materials, making them an increasingly compelling topic in energy storage research. By removing the host anode, these systems offer a simplified and potentially lower-cost manufacturing pathway. Their reduced volume and weight contribute to higher cell-level energy density, while the absence of a substantial amount of metallic lithium/sodium mitigates several safety risks [1,2]. The growing interest of the scientific community in this architecture is reflected in the consistent rise in publications over the last decade (Figure 1). To build on the dataset underlying this trend, a comprehensive set of keywords frequently used to describe these systems was employed—“anodeless,” “anode-less,” “anode less,” “anode free,” “anode-free,” “no excess lithium,” “no excess Li,” “lithium plating/stripping,” “Li plating/stripping,” “lithium nucleation,” and “Li nucleation”—combined with the term “battery.” These queries, conducted within article titles, abstracts, and keywords, formed the basis of a Scopus search performed on 3 December 2025. Of particular interest, the most pronounced growth phase in anode-less publications coincided with the period during which solid-state batteries experienced a significant surge in attention, suggesting that both strategies are increasingly viewed as complementary routes toward high-performance and safer lithium-based systems. The steady increase in scientific output over time further mirrors the industrial momentum toward adopting architectures that enhance safety while reducing environmental impact.

The present work was designed as a structured narrative review. While the Scopus search described above was used specifically to support the bibliometric overview shown in Figure 1, the literature discussed throughout this review was selected according to thematic relevance. Priority was given to studies directly related to anode-less/anode-free battery architectures, electrodeless concepts, interfacial phenomena, characterization methods, electrolyte-dependent design strategies, degradation mechanisms, and practical implementation challenges. Seminal contributions and recent representative studies were prioritized, whereas publications outside the direct scope of anode-less systems or not sufficiently related to the central themes of this review were not retained.

A growing body of review literature examining how the rigidity of solid interfaces fundamentally alters plating/stripping mechanisms and amplifies contact loss at the metal–electrolyte boundary has accompanied the transition toward anode-less solid-state batteries (ALSSBs). A recent synthesis detailed how in ALSSBs solid–solid discontinuities can generate void regions exceeding 50% of the active volume, requiring conformal ceramic buffer layers, such as doped LLZO variants, to reestablish both mechanical and electronic continuity [3]. The chemical specificity of sulfide electrolytes has motivated reviews dedicated to Li_2_S reduction processes and the redox instability of materials such as LPSCl, discussing emerging strategies including controlled precipitation of Ag to generate lithiophilic seeds and polymeric coatings that mitigate chelation leading to parasitic reactions [4,5]. Other analyses emphasize the architectural progression from conventional cells to bipolar designs capable of reducing parasitic resistance and allowing high-voltage stack architectures without compromising structural integrity [6,7]. Interface engineering at the current collector–solid-state electrolyte (SSE) contact is highlighted as a central technical axis, with studies discussing nanoscale texturing of the current collector to promote homogeneous ionic flux and control the growth direction of plated lithium [8]. From a more application-oriented perspective, reviews of pouch cells based on inorganic electrolytes, including garnet- and NASICON-type systems, highlight viable pathways toward reported energy densities above 300 Wh·kg^−1^, although such values should be interpreted within the assumptions and cell level specified in the cited work. These studies also emphasize the importance of mechanical control and stable operation under applied pressure [9]. In parallel, theoretical models have been developed to define the regime of operation under zero-excess conditions, establishing stability windows and acceptable current density thresholds to mitigate void formation and interfacial contact loss [10]. Complementary analyses centered on *operando* visualization illustrate how ionic transport pathways and plating morphologies evolve in inorganic and hybrid solid electrolytes, revealing local heterogeneities that cannot be captured through *ex situ* characterization [11].

The enthusiasm surrounding anode-less architectures has also extended to post-lithium systems, where sodium and potassium emerge as more abundant alternatives with potentially reduced logistical footprint. Reviews dedicated to sodium trace the evolution of ALSMBs since the mid-2010s, highlighting that the larger ionic radius of Na results in bulkier dendrites and poorer reversibility, thereby necessitating three-dimensional current collectors, such as Cu foams decorated with Sn, and concentrated electrolytes capable of achieving efficiencies above 99.5% [12]. In solid-state configurations, attention has focused on the limited Na^+^ conductivity of electrolytes such as NASICON and on the importance of external pressure to maintain interfacial conformity and suppress heterogeneous nucleation [13]. Other reviews explore the economic potential of these systems, noting that overall cost can be a fraction of that of Li-ion technologies, and highlight the use of metallic seeds to reduce the fraction of inactive sodium [14]. In the realm of interface engineering, research on strategies based on sodiophilic coatings emphasizes that chemical modification of the current collector surface is critical to achieving more than 300 stable cycles without significant active inventory loss [15]. More broadly, recent synthesis articles unify perspectives for Na, K, and even Zn, discussing alloy-mediated stripping mechanisms and delineating the cross-cutting challenges that persist in adapting the anode-less concept beyond lithium [16,17].

Given the growing number of recent review and perspective articles on anode-less batteries, a comparative summary is provided in Table 1 to clarify the scope of the existing literature and to better define the distinctive perspective of the present work.

**Figure 2:**
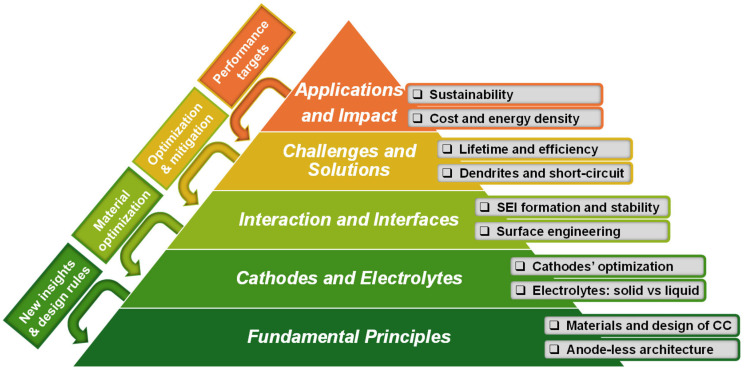
Pyramid of anode-less cell development: from fundamentals to applications.

As summarized in Table 1, recent overview articles have provided valuable insights into specific chemistries, interfaces, degradation mechanisms, and performance/improvement strategies. However, a broader perspective that systematically links fundamental principles, materials selection, interfacial phenomena, characterization methods, practical cell-level challenges, and future implementation pathways is still limited. To address this gap, the conceptual pyramid shown in Figure 2 was developed to organize the critical research and innovation topics in anode-less batteries. It progressively arranges the main levels of analysis, from fundamental principles and materials design, through interactions and interfaces, to the mitigation of complex operational barriers, ultimately culminating in applications and socioeconomic impact.

At the base of the pyramid are the fundamental principles, including anode-less architecture and current collector (CC) design, indispensable pillars for ensuring efficiency, safety, and viability. The next tier highlights the selection and optimization of cathodes and electrolytes, an essential step to maximize energy density and match the electrochemical compatibility of the whole system. Following this, the importance of interactions and interfaces is emphasized, particularly the formation and stability of the SEI (solid-electrolyte interphase), as well as advanced surface engineering strategies, critical aspects governing cell lifespan and performance.

The challenges and solutions level covers practical issues such as dendrite growth, short-circuits, and limitations in efficiency and durability, which frequently require iterative approaches and revisiting the structural and materials levels. Only after mitigating these challenges can the top of the pyramid be reached, aspiring to application-driven goals such as sustainability, competitive cost, and high energy density, criteria guiding the industrial translation of anode-less batteries.

It is important to highlight that the development process, symbolized by the lateral integration arrows in Figure 2, is intrinsically iterative: advances and obstacles encountered at a given level often require reevaluation and optimization of previous levels, thus promoting an integrated, dynamic, and continuous scientific evolution.

This conceptual pyramid supports the roadmap for this review article, acting as a guide for structured understanding, identification of research priorities, and definition of the most promising strategies to accelerate the maturity and practical implementation of anode-less batteries. This review presents a structured and integrative overview of the current state of anode-less battery research, encompassing both liquid- and solid-electrolyte systems. Electrodeless systems, which lack both anode and cathode materials, are also considered alongside anode-less cells to illustrate the progression from simpler to more complex architectures and to clarify how each component influences overall cell performance. By linking fundamental principles, interface engineering strategies, advanced characterization techniques, and practical cell-level challenges, the review provides a unified framework for understanding the development of anode-less batteries. It also identifies the major scientific and technological barriers that continue to limit practical implementation and outlines a research roadmap toward safer, higher-energy-density, and more industrially viable anode-less batteries. While solid-state anode-less batteries remain the main long-term implementation target, liquid and hybrid systems are discussed as mechanistic and developmental reference platforms that help contextualize interfacial behavior, metal deposition/stripping phenomena, and design principles relevant to the transition toward solid-state configurations.

## 2. Anode-Less Cells

### 2.1. Concept of Anode-Less Cells

The concept of anode-less (or anode-free) lithium-metal batteries (ALMBs) has been developed for over a decade and extensively investigated through experimental studies, which provided key demonstrations of this architecture. A foundational 2016 study [23] laid the groundwork for much of the subsequent research, demonstrating that metallic lithium can be deposited directly onto a current collector during the first charge, eliminating the need for a conventional preformed anode. This work emphasized the potential for higher energy density and a simplified negative electrode structure while also highlighting the principal technical challenges of this innovative battery design. Although the present discussion is centered primarily on lithium-based anode-less cells, it is important to note that the underlying concept was also demonstrated early in sodium systems, highlighting its broader relevance across alkali-metal chemistries. Early in that same year [24], it had been proved that plating Na from a Na^+^ glassy electrolyte on a copper current collector would revert a symmetric cell Cu/Na^+^ electrolyte/Cu into a 2.4–2.7 V cell.

**Figure 3:**
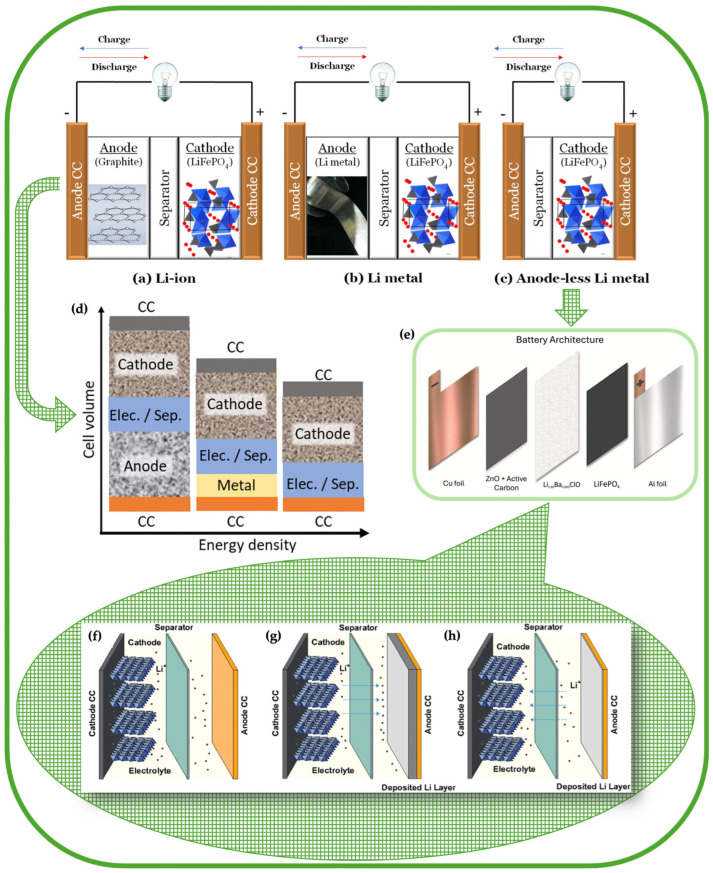
Battery cell configurations and fundamental operational mechanisms in anode-less/anode-free lithium-metal batteries. **Top**: Schematics of battery cell configurations: (**a**) lithium ion, (**b**) lithium metal, and (**c**) anode-less [26] (licensed under CC BY 4.0); **Center**: (**d**) Schematic comparison of cell volume for a conventional metal-ion battery, a metal battery (MB) with a metallic anode (e.g., Li, Na, K), and an anode-less metal battery (ALMB) utilizing the current collector (CC) as the nucleation site [17] (licensed under CC BY 3.0); (**e**) Detailed architecture of an ALMB in a pouch cell design [31] (licensed under CC BY 4.0); **Bottom**: Basic mechanism of anode-less lithium-metal batteries: (**f**) schematic of ALMBs, (**g**) charge process, and (**h**) discharge process [30] (licensed under CC BY-NC 3.0).

To place these early demonstrations in context, it is useful to contrast anode-less architectures with the conventional lithium-ion and metal-battery configurations from which they emerged. The development of electrochemical energy storage technologies has been dominated over recent decades by lithium-ion (Li-ion) batteries (Figure 3a). In this type of cell, the anode is typically composed of graphite, although silicon-based anodes have also attracted major interest because of their much higher theoretical capacity. However, silicon-based approaches still face important practical limitations related to lithium diffusion, interfacial instability, and large volume changes during cycling [25], while the cathode is commonly a lithium-metal oxide or phosphate (for example, NMC or LFP). During the charging process, lithium ions migrate from the cathode to the anode, intercalating into the graphite layers. In the reverse process, during discharge the ions return to the cathode. The electrons move to the same electrode as the ions through the external circuit, supplying electrical energy [26,27]. Despite their high technological maturity and widespread adoption in commercial applications, the capacity of Li-ion batteries is limited by the amount of lithium that can be intercalated into the cathode material, while the anode also restricts the practical use of high-capacity cathodes such as sulfur. While the theoretical capacity of graphite is 372 mAh·g^−1^, sulfur offers a much higher theoretical value of ~1660 mAh·g^−1^, which explains the strong interest in Li–S systems for next-generation high-energy batteries, despite their well-known materials and interfacial challenges [28].

Lithium/sodium metal batteries (MBs) have emerged as a promising alternative to overcome this limitation (Figure 3b). In these systems, the anode is composed of metallic lithium/sodium, which enables achieving a significantly high theoretical specific capacity (Li: 3860 mAh·g^−1^ and Na: 1166 mAh·g^−1^). Additionally, a low reduction potential (Li/Li^+^: −3.04 V and Na/Na^+^: −2.71 V vs. standard hydrogen electrode (SHE) and −1.40 eV and −1.73 eV vs. electrons at rest in vacuum) and a small ionic radius justify its applicability in various devices [2,29]. However, the use of metallic lithium/sodium is associated with serious challenges: these metals have high chemical and electrochemical reactivity, whereby they react easily with the electrolyte, causing electrolyte consumption, corrosion, dendrite growth, and formation of irreversible solid-electrolyte interface (SEI) passivation layers [2,27]. Thus, the presence of excess lithium/sodium in the anode of the MBs poses safety hazards and increases their production cost, as well as a decreased efficiency, actual specific capacity, and volumetric capacity [26,30].

To mitigate such limitations, ALMBs have been investigated (Figure 3c). This configuration allows for a reduction in cell volume compared to conventional metal-ion or standard metal batteries (MBs), as the cell is assembled without a preformed active anode. Instead, only a current collector (typically copper) functions as the nucleation site (Figure 3d). However, the electrolyte should compensate for the loses in mobile alkali ions. Otherwise, the capacity of the cell decreases. The practical implementation of this concept can be seen in the detailed architecture of an ALMB when integrated into a pouch cell design (Figure 3e,f). During the first charge, lithium is extracted from the cathode as Li^+^ and electrochemically deposited onto the collector thorough the electrolyte, forming the Li^0^ anode *in situ* and *in operando* (Figure 3g,h). This simplified design eliminates the need to package metallic lithium, reduces the weight and volume of the cell, and increases both gravimetric and volumetric energy density. Furthermore, the concept is compatible with the already established production technology of lithium-ion batteries, which supports its industrial feasibility [26,30,31,32].

The operation of the batteries depends on the storage of ions in the cathode. Consequently, it is associated with the previous step, i.e., during the electrodeposition of the ions in the anode current collector. Although lithium/sodium foils or other anodes are not used, the presence of lithium/sodium ions is required at the cathode (for example, with LiFePO_4_). After charging, the formation of a temporal anode takes place with the Li transported from the cathode to the electrolyte, and this is the only source available for the discharge step in traditional anode-less battery besides the lithium in the electrolyte [2]. Therefore, after the charging step the battery without anode functions as a lithium/sodium metal battery, but with limited material. Columbic efficiency is generally used as an indicator of Li/Na utilization because its value reflects the stability and reversibility of the anode-less cell cycle [2].

In simpler terms, discharge in an anode-less battery is driven by the spontaneous tendency to reduce the electrochemical potential difference between the two electrodes. Electrons move through the external circuit from the negative to the positive electrode, while mobile ions (e.g., lithium ions) are transported through the electrolyte toward the cathode side. At the interfaces, electrical double layers arise because of the chemical potential difference between the electrolyte (insulator) and each electrode (conductors). In fact, the negative electrode has higher absolute chemical potential than the positive electrode. Therefore, the electrons are spontaneously driven from the negative to the positive electrode (through the external circuit) and so are the ions, although they diffuse within the electrolyte [33]. Thus, when the electrochemical potentials are equalized, the chemical potential difference will equal ${µ}_{\left(-\right)}-{µ}_{\left(+\right)}=z_{i}F\left(\phi_{\left(+\right)}-\phi_{\left(-\right)}\right)$, where $z_{i}$ is the valency of the mobile ion ($z_{{Li}^{+}}=\;$1), F is Faraday’s constant, $\phi_{\left(+\right)}$ is the surface potential of the cathode, and $\phi_{\left(-\right)}$ is the correspondent for the anode, meaning that as the electrons migrate from the negative to the positive electrode, the difference in chemical potentials (${µ}_{\left(-\right)}-{µ}_{\left(+\right)}$) decreases and consequently the potential difference $∆V=\phi_{\left(+\right)}-\phi_{\left(-\right)}$. It is emphasized that the creation of double-layer capacitors at the interfaces, with total energy q$∆V=q{(\phi }_{\left(+\right)}-\phi_{\left(-\right)})$ where q is the accumulated charge, happens to compensate for the chemical potential differences between the materials when one of the materials involved is an insulator and may not exchange electrons [34]. However, as the chemical potentials at the negative electrode/electrolyte interface spontaneously equalize during discharge, the previously accumulated double-layer charge is no longer needed, and Li+ diffuses through the electrolyte toward the positive electrode. The electrons reach the cathode tunnel to the electrolyte’s surface. There, they reduce the Li^+^, and consequently the Li atoms may diffuse more freely as a neutral atom back to the cathode by the Fick’s law effect, since there is a lower concentration of Li on the cathode side [35]. Then a lithium-rich phase starts to form. When the charged lithium poor phase and the discharged lithium rich phase equilibrate, the chemical potentials of the phases in equilibrium do not change and a voltage plateau with ∆V=constant is formed. If the lithium poor phase is FePO_4_, the equilibrium happens between FePO_4_ and LiFePO_4_. The potential difference ∆V decreases abruptly when FePO_4_ runs out and LiFePO_4_ becomes the sole active cathode phase. A step drop in the potential difference is observed, because the chemical potential of LiFePO_4_ increases decreasing the difference to the anode’s until the cutoff potential is reached. It is not standard practice to allow the battery to discharge to zero, as spurious reactions may occur [36,37].

The fundamental concepts of anode-less lithium-metal batteries continue to guide current research, with growing emphasis on optimizing the surface of the negative current collector to promote uniform lithium nucleation. Recent studies have demonstrated that careful surface conditioning [26], as well as the design of in-series configurations to enhance overall output [38], can significantly improve the performance and cyclability of both conventional and all-solid-state anode-less cells. Moreover, interface engineering strategies, including multisite nucleation supported by elastic networks [39], and dual-seed designs [40] have been shown to suppress dendrite growth and promote homogeneous lithium plating. Additional approaches, such as ZnF_2_ surface modification for *in situ* structural regulation [41] and the use of 3D copper foam current collectors with lithiophilic Cu_2_O and LiF-rich interfacial layers [42], further highlight the diversity of solutions under exploration. Building on these developments, the following section discusses the key advantages of anode-less cells and the remaining technical challenges that must be addressed to fully exploit their potential.

### 2.2. Advantages and Challenges

Anode-less batteries (ALBs) have recently gained significant attention as a promising next-generation energy storage concept, offering a simplified architecture, higher gravimetric and volumetric energy densities, and potential reductions in weight and manufacturing cost by eliminating the need for a preformed metallic anode [30,43,44,45]. Beyond these intrinsic advantages, novel design strategies such as bipolar configurations in solid-state batteries enable compact device architectures with enhanced volumetric performance [7]. The versatility of the ALB concept has also been demonstrated in post-lithium chemistries [17] and sodium-based systems [14], expanding its scope beyond conventional Li-ion technology. Even in sulfide-based solid-state batteries, which present significant interfacial challenges, the anode-less approach holds potential for substantial breakthroughs once these issues are mitigated [4,46].

These theoretical advantages are increasingly supported by experimental evidence, where different approaches have been employed to validate the practical feasibility of the anode-less concept. Sacrificial salts have been shown to enable anode-less prototyping without the requirement of excess metallic lithium [47], while lightweight lithiophilic interfaces have demonstrated improvements in coulombic efficiency and cycling stability [48]. In the solid-state domain, stable operation has been achieved at room temperature and ambient pressure [49], and conditioning strategies of current collectors have been proposed to control lithium plating and stripping [26]. Additional interfacial engineering, such as the use of directionally grown Cu (220) current collectors, has enabled long-term cycling stability with high coulombic efficiency [50].

Finally, experimental demonstrations in alternative systems further highlight the flexibility of the anode-less principle. Intermetallic Na–Sb–Te alloys have allowed sodium-metal cycling at 100% depth of discharge under anode-less conditions [51], while hybrid Na–CO_2_ batteries integrating sodium harvested from seawater illustrate both the multifunctionality and sustainability potential of anode-less designs [52]. Together, these advances confirm that anode-less batteries not only present a theoretical pathway toward higher energy density and structural simplification but also show concrete experimental progress, reinforcing their potential as a viable next-generation energy storage technology.

Despite these advantages, anode-less batteries still face severe challenges that currently limit their practical deployment. Reviews consistently emphasize that the absence of an initial lithium reservoir renders these systems extremely sensitive to the irreversible loss of active lithium, which occurs through side reactions, SEI formation, and the accumulation of electrochemically inactive “dead Li” [22,46,53]. Therefore, ALMB cycle life is dramatically shortened unless extremely high coulombic efficiencies (>99.9%) are achieved. Another critical challenge lies in ensuring uniform lithium plating and stripping: inhomogeneous deposition promotes dendrite growth and localized current hot spots, which in turn can trigger internal short circuits [30,43]. Moreover, the stability of the current collector–electrolyte interface is a major barrier, since repeated expansion and contraction during cycling often leads to contact loss, interfacial degradation, and continuous electrolyte decomposition [4,7]. These issues are intensified in solid-state systems, where the mechanical rigidity of the electrolyte makes it even harder to maintain intimate and stable interfaces.

Experimental studies provide concrete evidence of these limitations. Even when sacrificial salts are employed to compensate for the initial lithium deficiency, side reactions still reduce the overall efficiency and limit long-term stability [47]. Interface engineering has been shown to mitigate dendrite growth and enhance plating uniformity, but stability remains constrained to relatively short cycle numbers [48]. In solid-state architectures, attempts to operate at room temperature and low pressure revealed persistent interfacial resistance growth and limited reversibility [49], while conditioning strategies for current collectors only partially stabilized lithium deposition [26]. Likewise, nanoscale surface modifications, such as the fabrication of Au nanopatterns or the preferential growth of Cu (220) facets, improved coulombic efficiency and delayed failure, but did not eliminate the intrinsic lithium loss problem over extended cycling [50,54]. Similar trends are observed in sodium-based systems, where intermetallic hosts or hybrid chemistries reduce, but do not suppress the challenges of sodium loss, dendrite formation, and unstable interfaces [51,52].

Taken together, these findings indicate that while the conceptual advantages of anode-less batteries are clear, their practical realization is still hindered by lithium (or sodium) inventory depletion, nonuniform metal deposition, dendrite growth, and unstable interfaces. Recent comprehensive analyses demonstrate that lithiophilic seed interlayers (Mg, Ag, Sn) can significantly improve deposition homogeneity and cycling stability across both liquid and solid-state electrolytes, achieving high coulombic efficiencies in practical anode-less cells [55]. Addressing these fundamental issues through such advanced interfacial engineering remains essential for unlocking the full potential of this technology.

### 2.3. Characterization and Analysis Techniques

The performance and practical deployment of anode-less cells depend on a precise understanding of electrochemical, interfacial, structural, and thermal processes. Unlike conventional cells, these systems face additional challenges related to lithium plating/stripping, interfacial stability, and morphological evolution during cycling. Comprehensive characterization is therefore indispensable, not only to probe intrinsic material properties but also to capture dynamic processes that dictate efficiency and durability. For clarity, the most relevant techniques are grouped into five categories: (*i*) electrochemical analyses, (*ii*) interfacial potential mapping, (*iii*) structural and chemical analyses, (*iv*) morphological and evolution studies, and (*v*) thermal analyses.

#### 2.3.1. Electrochemical Analyses: Charge–Discharge Cycling, PEIS, CV, GITT

Electrochemical analyses are essential for assessing the performance of anode-less cells, providing information from overall cycling behavior to detailed kinetic parameters. Charge–discharge cycling evaluates capacity, coulombic efficiency, and long-term stability, potentiostatic electrochemical impedance spectroscopy (PEIS) probes internal resistance and interfacial properties, cyclic voltammetry (CV) explores the redox processes and reversibility, and the galvanostatic intermittent titration technique (GITT) quantifies ionic diffusivity and slow kinetic phenomena, offering a comprehensive view of electrochemical behavior.

Charge–discharge cycling remains the primary tool to evaluate the reversibility and stability of anode-less cells, enabling the identification of degradation pathways and the validation of interfacial engineering strategies. For instance, the incorporation of lithiophilic seeds in both liquid and solid-state electrolytes demonstrated that galvanostatic cycling can clearly differentiate between stable Li nucleation and progressive interfacial degradation [55]. Similarly, electrochemical conditioning of solid-state anode-less cells enhanced performance and reduced polarization during initial cycles [26]. Cycling tests have also been crucial to validate innovative material architectures, including chlorocatechol-cross-linked polymer separators [56] and elastic network frameworks enabling multisite Li nucleation [39]. Advanced approaches have extended cycling studies to more complex electrode architectures, such as lithio-amphiphilic nanobilayers that promote high energy density under low stack pressure [57], dual-metal interlayers tailored to suppress dendritic growth [58], and dual-seed strategies that stabilize Li plating/stripping across extended cycling [40]. In parallel, three-dimensional copper collectors [59] and free-standing graphene films decorated with lithiophilic particles [60] have demonstrated improved cycling stability by homogenizing nucleation and guiding Li deposition, with the former delivering about 100 mAh·g^−1^ and 99.9% CE after 50 cycles and the latter showing ~60% capacity retention after 120 cycles at 1.93 mA·cm^−2^ in NMC811-based anode-less cells. More exotic designs, such as lithiated graphene current collectors [61], fluorinated porous frameworks [62], and bilayer separators that ensure uniform Na deposition [63], have further confirmed the centrality of cycling tests in evaluating interfacial stability across different chemistries. Moreover, *operando*-compatible studies have revealed that electro-chemomechanical coupling during cycling governs Li plating reversibility and interfacial degradation [64]. Collectively, these works demonstrate that charge–discharge cycling is not only a benchmark for assessing reversibility and efficiency but also a powerful diagnostic tool to validate interfacial design strategies across diverse anode-less architectures.

PEIS is often combined with cycling to investigate changes in interfacial resistance and charge-transfer dynamics. Direct evidence of its relevance in anode-less systems includes studies on electrochemical conditioning of solid-state cells, where impedance spectra revealed a progressive reduction in interfacial resistance during the initial cycles [26], as well as in-series all-solid-state anode-less configurations, in which PEIS identified ionic transport limitations between sub-cells and interfacial degradation in stacked arrangements [38]. Additionally, *in situ* polymerizing electrolytes demonstrated that PEIS effectively captures the evolution of interfacial resistance during the liquid-to-solid transformation, highlighting its applicability to monitor dynamic interfacial processes [65].

CV complements cycling data by monitoring redox stability and interfacial reactions. Conditioning of solid-state anode-less cells was monitored via CV, revealing the formation of stable interphases [26], while *in situ* polymerizing electrolytes exhibited distinct oxidation peaks associated with trimethylsilyl phosphate (TMSP)-derived polymeric phases [65]. Furthermore, CV analyses were also presented in in-series solid-state anode-less cells, allowing evaluation of redox stability and interfacial reactions in more complex arrangements [38]. In addition, CV has been widely employed to assess the electrochemical stability of novel solid electrolytes, as exemplified by studies on ferroelectricity-based systems [66], highlighting its versatility in probing interfacial and redox processes beyond conventional anode-less configurations.

GITT, although less commonly used, provides quantitative insights into ionic diffusion and kinetic limitations. Elastic network frameworks improved Li^+^ transport, as evidenced by diffusion coefficients extracted via GITT, correlating with enhanced cycling stability [39]. Dual-seed nucleation strategies also showed faster ionic transport and reduced polarization [40]. At larger scales (Ah level), prelithiated separators facilitated more homogeneous diffusion pathways, as confirmed by GITT [67]. Importantly, GITT is not limited to lithium systems: in anode-less Zn batteries, studies confirmed that interfacial engineering stabilizes Zn^2+^ transport, highlighting the versatility of this technique across different chemistries [68].

**Figure 4:**
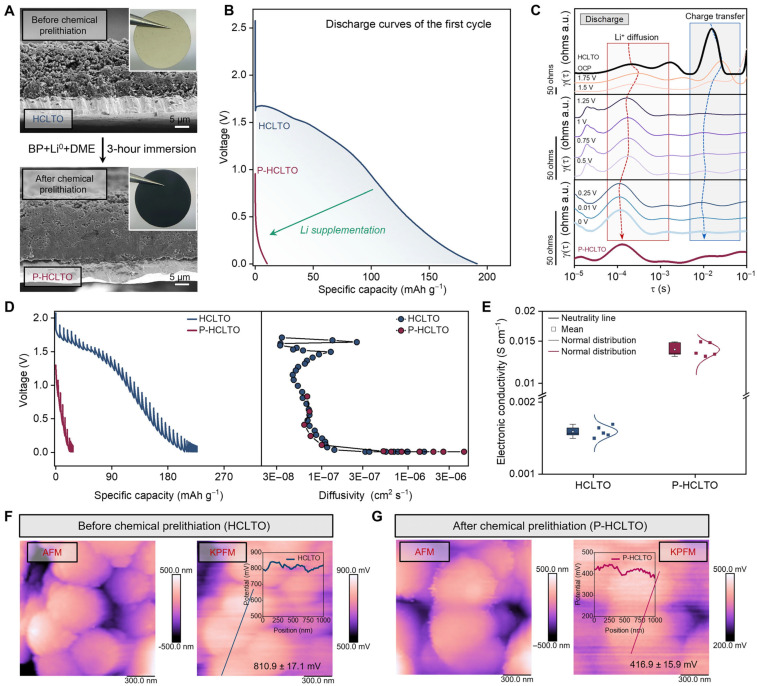
Chemical prelithiation and electrochemical/interfacial characterization of HCLTO interlayers. (**A**) Cross-sectional SEM images and surface digital images (insets) of the HCLTO interlayer before chemical prelithiation and of the chemically prelithiated interlayer (P-HCLTO) after 3 h immersion in BP + Li^0^ + DME. (**B**) First-cycle galvanostatic discharge curves of Li||Cu cells employing HCLTO and P-HCLTO interlayers, highlighting the effect of Li supplementation. (**C**) Distribution of relaxation time (DRT) analysis of EIS data for HCLTO and P-HCLTO, showing the evolution of Li^+^ diffusion and charge-transfer processes during discharge. (**D**) Galvanostatic discharge profiles (left) and the corresponding evolution of the diffusion coefficient derived from GITT analysis (right). (**E**) Electronic conductivity of HCLTO and P-HCLTO measured by the four-probe method. (**F**,**G**) AFM topography and the corresponding KPFM surface-potential maps of the interlayers before (**F**) and after (**G**) chemical prelithiation [69] (licensed under CC BY-NC 4.0).

Figure 4 compiles results from different studies and material systems, illustrating how these electrochemical techniques are essential for elucidating the operational behavior of solid-state cells [26,69].

#### 2.3.2. Interfacial Potential Mapping: SKP, KPFM

Mapping interfacial potentials is essential to unravel charge distribution and surface-potential evolution at the electrode–electrolyte interface in anode-less systems. Scanning Kelvin probe (SKP) provides macroscopic mapping of surface potentials and has been employed to monitor *in operando* work-function shifts in hybrid heterocells [70], differentiate materials’ energy-level alignment [71], and track synergistic lithium alloying and plating behavior in engineered 3D current collectors [72]. These studies demonstrate the ability of SKP to resolve macroscopic potential variations and link them to interfacial reactivity, charge transport, nucleation processes, and long-term stability.

Kelvin probe force microscopy (KPFM) provides nanoscale maps of contact potential difference (CPD), allowing high-resolution visualization of interfacial surface-potential heterogeneities that dictate Li nucleation and plating. For instance, combined KPFM and electrochemical AFM investigations have correlated surface-potential variations with lithium nucleation and growth dynamics, revealing how electrode potential governs early deposition pathways [73]. *Operando* KPFM has further enabled dynamic mapping of potential redistribution in functioning solid-state cells during cyclic voltammetry, exposing local electrochemical asymmetries between charge and discharge [74]. Cross-sectional *in situ* KPFM has been employed to image buried potential gradients in charged solid-state Li-ion cells, highlighting the presence of nonuniform internal fields that can trigger localized degradation [75]. More recently, *operando* KPFM studies on garnet-based electrolytes visualized Galvani potential drops at grain boundaries during Li plating, directly linking electron accumulation and interfacial instabilities to dendrite nucleation [76].

**Figure 5:**
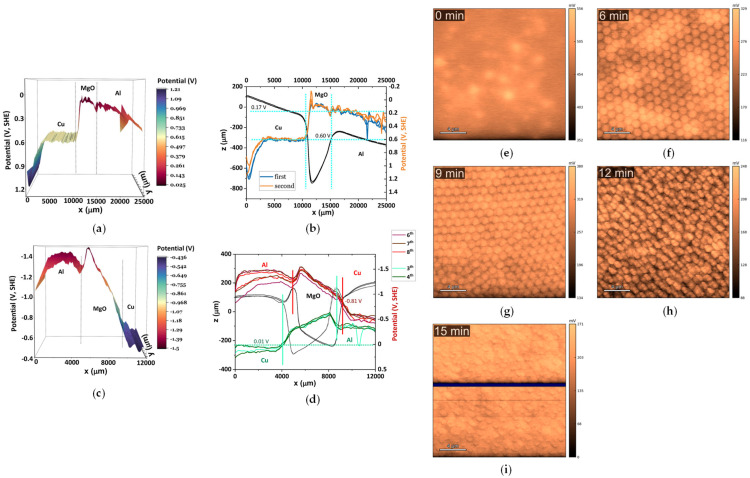
Comparative representation of surface-potential analyses using scanning Kelvin probe (SKP) and Kelvin probe force microscopy (KPFM). (**a**–**d**) Surface topography and surface chemical potentials of an Al–MgO–Cu cell, highlighting the distinct equalization energies of the Cu–MgO, MgO–Al, and Al–MgO heterojunctions, as well as the band-bending response depending on the electrode connected to the SKP [71] (licensed under CC BY 4.0). (**e**–**i**) KPFM-derived maps of the surface potential of Cu current collectors subjected to different etching durations (0–15 min), with dark-blue masked regions corresponding to artefacts associated with local charge accumulation and excluded from the averaged potential analysis [77] (licensed under CC BY 4.0).

Figure 5 provides a comparative overview of surface-potential measurements obtained by SKP and KPFM, highlighting how different interfaces and surface treatments influence the electronic landscape of current collectors [71,77].

Together, SKP and KPFM provide complementary perspectives: SKP allows global, *in operando* monitoring of work-function shifts over large areas, while KPFM resolves nanoscale potential variations at buried or evolving interfaces. Their combined use has proven invaluable for elucidating the interplay between interfacial energetics, nucleation mechanisms, and long-term stability in anode-less solid-state and hybrid battery systems.

#### 2.3.3. Structural and Chemical Analyses: XRD, XPS, ToF-SIMS, TEM, LIBS, *Operando* Techniques

Structural and chemical characterization techniques are essential for understanding the evolution of materials and interfaces in anode-less batteries. Combining *ex situ* and *operando* methods allows comprehensive analysis of processes occurring during charge and discharge cycles.

X-ray diffraction (XRD) provides structural information, enabling monitoring of cathodes’ phase transitions and lattice variations during cycling of ALMBs. *In operando* studies have revealed structural evolution and transition dynamics under fast charging conditions [78]. Additionally, Li–Ag alloy phase evolution in anode-less cells under both liquid and solid electrolytes has been characterized by diffraction techniques, providing insights into phase stability and interfacial evolution [79]. More recently, XRD has been applied to advanced anode-less designs, evidencing the reversible formation and dissolution of Li and alloy phases in dual-seed strategies [40] and confirming the crystallinity and phase purity of engineered lithiophilic interlayers that stabilize Li plating and stripping [48].

X-ray photoelectron spectroscopy (XPS) is a key technique for elucidating the chemical composition and evolution of interphases in anode-less batteries. By providing surface-sensitive information on elemental states and bonding environments, XPS enables identification of SEI constituents and decomposition products that critically influence cycling stability. In anode-less all-solid-state batteries, XPS has been used to confirm the impact of personalized interfacial wetting layers on the chemical stability of the Cu current collector, correlating interfacial chemistry with improved lithium nucleation and reversibility [80]. Complementary studies highlight the ability of XPS to resolve SEI composition and evolution in both lithium-metal and anode-less systems, revealing the role of inorganic and organic species in stabilizing or destabilizing the interface [21]. Moreover, in solid-state anode-less cells, XPS has been crucial in identifying conditioning-induced modifications that promote more uniform interphases and suppress parasitic reactions [26]. Extending beyond lithium, XPS has also unveiled the chemistry of high-entropy SEI layers that enhance interfacial robustness in sodium anode-less batteries [81]. Finally, degradation pathways in Li anode-less cells, including electrolyte breakdown and accumulation of resistive interphases, have been directly probed through detailed XPS analyses, providing mechanistic insights into performance fading [82]. However, because XPS is intrinsically surface-sensitive, its limited probing depth may hinder the analysis of thick, heterogeneous, or strongly depth-dependent interphases.

Time-of-flight secondary ion mass spectrometry (ToF-SIMS) provides high-resolution spatial and chemical mapping of electrode surfaces and interphases, making it a powerful tool for elucidating interfacial phenomena in anode-less batteries. By detecting the distribution of metal ions, decomposition products, and additives at the nanoscale, ToF-SIMS enables direct visualization of heterogeneities in lithium, sodium, and potassium deposition. In lithium anode-less cells, the technique has been employed to investigate the effect of lithiophilic seed layers and dual-metal interlayers on Li nucleation and growth, revealing more uniform plating and reduced dendritic structures [55,58]. Similarly, strategies such as near-surface Li-ion irrigation and lithio-amphiphilic nanobilayers have been characterized by ToF-SIMS to confirm enhanced deposition homogeneity and interface stabilization [57,83]. Beyond lithium, ToF-SIMS has proven essential for sodium and potassium anode-less systems, allowing the mapping of hybrid SEI layers, high-affinity interfaces, and deposition uniformity, thereby elucidating mechanisms that strengthen long-term stability and high coulombic efficiency [84,85,86,87].

Transmission electron microscopy (TEM) is a powerful technique to resolve interfacial structures, deposition morphology, and phase transformations in anode-less batteries at the nanoscale. It provides direct evidence of lithium or sodium nucleation pathways, interlayer stability, and the evolution of interphases during cycling. In solid-state configurations, TEM has been employed to unravel the role of metallic interlayers in stabilizing Li plating and stripping, revealing controlled nucleation and suppressed void formation [88]. Similarly, engineered host structures such as Sn-decorated doped carbon fibers and electron-deficient carbon collectors have been imaged to demonstrate homogeneous Li deposition and reduced interfacial stress [89,90]. TEM has also confirmed the uniform distribution and crystallinity of lightweight, lithiophilic interlayers and advanced engineered coatings designed to guide Li nucleation and suppress dendrites [48,91]. Furthermore, conversion-type anode-less systems based on metal fluorides have been studied by TEM to visualize nanoscale phase formation and structural changes upon cycling [92]. Extending beyond lithium, TEM has revealed the morphology and interfacial characteristics of MOF-derived nanocomposites used as high-affinity current collectors in sodium anode-less batteries [93]. Finally, structural insights into oriented copper substrates and liquid metal coatings have been obtained, demonstrating how crystallographic control and interfacial wetting govern Li deposition uniformity and cycle stability [50,94]. Despite its exceptional spatial resolution, TEM (particularly *in operando* TEM) may be affected by electron-beam-induced artifacts, and the simplified sample geometries typically required are not always fully representative of bulk cell behavior.

**Figure 6:**
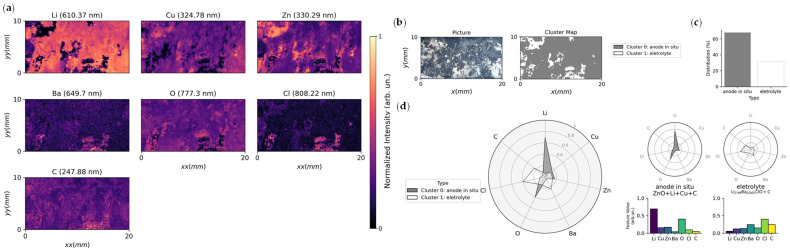
Combined LIBS-based surface analysis of solid-state anode-less cells. (**a**) LIBS elemental distribution maps for Li (610.37 nm), Cu (324.78 nm), Zn (330.29 nm), Ba (649.7 nm), O (777.3 nm), Cl (808.22 nm), and C (247.88 nm), each normalized to its maximum signal intensity [31] (licensed under CC BY 4.0). (**b**) Negative current collector alongside (**c**,**d**) clustering analysis segmented spatial distribution of the identified clusters, quantitative contribution of each compound, and radar chart of feature weights supporting the interpretation of cluster assignments [31] (licensed under CC BY 4.0).

Laser-induced breakdown spectroscopy (LIBS) has recently emerged as a promising tool for surface and interfacial analysis in anode-less batteries, offering rapid and spatially resolved chemical characterization. The technique is inherently destructive at local measurement spots, as laser ablation generates a plasma plume from which the elemental composition is derived. Despite this, LIBS enables depth profiling and mapping over larger areas, providing valuable insights into elemental distributions across interfaces. Its applicability has been demonstrated in solid-state anode-less systems, where LIBS was used to probe interfacial chemistry and detect compositional changes during cycling [31]. Broader perspectives on the integration of LIBS into the analytical toolbox for solid-state anode-less batteries highlight its complementarity with established methods and its potential for rapid diagnostics [95]. In practical studies, LIBS has also been applied to investigate conditioning in solid-state anode-less cells, linking interfacial modifications with enhanced cycling stability [26]. Although still scarcely explored across different anode-less battery configurations, LIBS has been recognized as a promising approach to complement surface and bulk, sensitive techniques, bridging gaps in the characterization of next-generation anode-less electrodes [96]. Figure 6 illustrates spatially resolved LIBS analysis and subsequent clustering evaluation applied to a solid-state anode-less cell, providing complementary insight into the elemental distribution and surface composition of the negative current collector. It is highlighted that LIBS detects lithium directly, conversely to scanning electron microscopy/energy-dispersive X-ray spectroscopy (SEM–EDX).

*In operando* techniques provide unique insights into structural, chemical, and morphological changes in anode-less batteries during real-time operation, capturing phenomena that cannot be fully resolved by *ex situ* analyses. *In operando* XRD has been widely employed to track phase transitions, lattice changes, and alloy formation dynamics in lithium, sodium, and potassium anode-less cells [88,97,98]. These studies reveal the real-time structural evolution of the electrodes and interlayers, enabling direct correlation between phase transformation pathways, alloying–dealloying reversibility, and the onset of degradation processes such as mechanical instability, interfacial loss of contact, or irreversible phase accumulation. *In operando* TEM has been used to directly visualize Li nucleation, growth, and dissolution, as well as the evolution of interlayers and coatings during cycling [88,94]. TEM studies reveal morphological evolution at the nanoscale, dendrite formation, and interfacial degradation, providing complementary structural information to XRD. *In operando* spectroscopic techniques, including XPS and Raman spectroscopy, allow monitoring of interphase formation and chemical evolution of solid-electrolyte interphases (SEI) in real time, correlating these transformations with electrochemical performance. Specifically, *in situ/operando* Raman has been applied to lithium anode-less batteries to study the effect of polydopamine-coated substrates on Li nucleation and interface stabilization [99]. It has also been employed with lithium–sulfur systems for tracking polysulfide species and guided Li nucleation [100], to sodium anode-less batteries for high-entropy SEI formation [81], and to oriented copper collectors for probing interfacial chemical dynamics [50]. Collectively, these *in operando* approaches are essential for understanding the complex interplay between electrode materials, interfacial layers, and ionic transport in anode-less batteries. By combining structural, morphological, and chemical insights, *in operando* techniques guide the rational design of more stable, high-performance anode-less systems, bridging gaps that static or *postmortem* analyses cannot fully resolve.

#### 2.3.4. Morphological Studies: SEM–EDX, AFM, in *Operando* Tomography

Morphological analyses are essential to capture both surface and bulk evolution driven by lithium plating and stripping in anode-less batteries. SEM, often combined with EDX, enables visualization of surface topography and elemental distributions, providing insights into interfacial degradation and electrodeposition pathways, though as mentioned previously, it may not detect. Atomic force microscopy (AFM) extends this characterization to the nanoscale, revealing fine details of surface roughness, nucleation sites, and interphase formation. Complementarily, *in operando* X-ray tomography allows three-dimensional monitoring of morphological changes throughout cycling, offering real-time correlation between structural evolution and electrochemical performance. Together, these techniques establish a comprehensive framework for understanding and controlling interfacial processes in anode-less systems.

SEM is often combined with EDX, and is a keystone technique for probing morphological evolution and elemental distributions in anode-less batteries, although SEM–EDX does not detect lithium directly, as mentioned before. SEM–EDX analyses enable visualization of alkali-metal plating/stripping behavior, interphase degradation, and the impact of interface engineering strategies. For instance, the distribution of silver particles in carbon nanocomposite interlayers has been directly correlated with lithium nucleation and plating patterns through SEM–EDX imaging [101]. Similarly, interfacial degradation processes leading to corroded but reactive interphases have been characterized by microstructural inspection, shedding light on stability under extended cycling conditions [102]. SEM–EDX has also been employed to evaluate conditioning in solid-state anode-less cells, highlighting interfacial smoothing and compositional redistribution as contributors to improved cycling behavior [26]. Beyond lithium systems, morphological studies on seawater anode-less batteries have revealed Na deposition features and corrosion phenomena, emphasizing the broader applicability of these methods [103]. In addition, SEM–EDX has proven crucial for assessing surface modifications of current collectors [104], validating the effectiveness of nitrate-preplanted Li composite layers [105], and confirming the formation of black phosphorene conductive layers under external pressure [106]. Collectively, SEM–EDX provides direct microstructural and compositional evidence of interfacial processes, supporting the design of stable architectures for next-generation anode-less systems.

AFM provides nanoscale insights into surface morphology, enabling direct observation of nucleation sites, roughness evolution, and interfacial transformations in anode-less batteries. This technique has been employed to investigate interfacial modifications, such as ZnF_2_ coatings, which regulate lithium deposition and stabilize cycling behavior [41]. In lithium–sulfur anode-less systems, AFM revealed how hybrid interfacial modulation guides Li nucleation while mitigating polysulfide-induced inhomogeneities [100]. *Operando* AFM has also been crucial for clarifying the allometric growth and early dissolution of Zn anodes, capturing nucleation dynamics with nanometer resolution [107]. Moreover, AFM analyses confirmed the morphological impact of engineered collectors and interphases, including phosphorized 3D Cu substrates [108], garnet-based composite electrolytes [109], hydrophobic ion barriers inducing Zn (002) orientation [68], intermetallic Na-seeding layers [110], and polyaniline-modified Cu collectors [111]. Collectively, AFM serves as a unique probe to resolve interfacial evolution at the nanoscale, complementing SEM–EDX observations and guiding the rational design of stable architectures for diverse anode-less chemistries.

**Figure 7:**
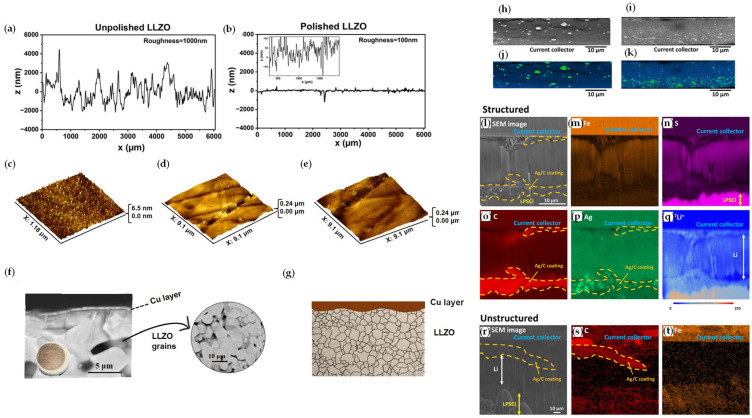
Comparative interfacial characterization strategies for anode-less solid-state batteries. Linear profilometry of LLZO pellets—(**a**) unpolished and (**b**) polished; AFM topography of (**c**) Cu/Si, (**d**) LLZO, and (**e**) Cu/LLZO, cross-sectional SEM of (**f**) 600 nm sputtered Cu on LLZO, and (**g**) schematic of Cu-layer attachment to LLZO grains [112] (licensed under CC BY 4.0). SEM cross sections of (**h**) unstructured and (**i**) structured Ag/CB interlayers after compression at 500 MPa; corresponding Ag/C EDS maps for (**j**) unstructured, and (**k**) structured films [101] (licensed under CC BY 4.0). Cross-sectional analysis of stainless steel/current collector/Ag–CB/LPS regions under a single charge at 2.5 mA·cm^−2^: (**l**) SEM of structured interlayer; (**m**–**p**) EDS maps of S, C, Ag, and Fe; (**q**) ^7^Li^+^ SIMS map; (**r**) SEM of unstructured interlayer; (**s**,**t**) EDS maps of C and Fe [101] (licensed under CC BY 4.0).

Figure 7 illustrates representative experimental results obtained using techniques applied to the study and development of anode-less cells, highlighting how multiscale characterization informs interface design and understanding [101,112].

*In operando* X-ray tomography provides unique three-dimensional insights into the structural evolution of anode-less batteries during cycling, enabling real-time observation of metal deposition, interfacial dynamics, and mechanical degradation. In zero-excess lithium all-solid-state batteries, *operando* tomography has shown that interfacial nucleation nanolayers play a critical role in suppressing mechanical failure, preventing crack formation during cycling [113]. Similarly, the combination of *operando* X-ray computed tomography with machine learning has been used to detect and quantify lithium plating dynamics, providing detailed maps of spatial inhomogeneities and growth patterns [114]. Beyond direct anode-less studies, methodological advances such as X-ray nanotomography for quantitative material analysis [115], X-ray diffraction computed tomography for reaction and rate heterogeneity [116], and multiscale coupling of tomography with diffraction or pair distribution function analyses [117,118] highlight the versatility and growing capabilities of *operando* tomography techniques across different battery chemistries. Collectively, these studies demonstrate how *operando* tomography can directly link three-dimensional structural evolution to electrochemical performance, offering critical guidance for the design of more stable anode-less systems.

#### 2.3.5. Thermal Analyses: In *Operando* Calorimetry, DSC, ARC

Thermal analyses are essential for understanding the safety and thermal behavior of anode-less cells, where uncontrolled heat generation can induce instability due to the lack of excess lithium and the fragile nature of interphases. *In operando* calorimetry enables real-time monitoring of heat generation associated with plating/stripping and parasitic reactions, directly correlating thermal signatures with electrochemical cycling. Differential scanning calorimetry (DSC) provides quantitative insights into exothermic and endothermic processes, showing phase transitions, interfacial decomposition, and SEI-related reactions. Accelerating rate calorimetry (ARC) further assesses thermal runaway risks under extreme operating conditions, offering critical guidance for thermal management strategies and the safe deployment of anode-less batteries in both liquid- and solid-state electrolyte systems.

*In operando* calorimetry offers a powerful approach to quantifying heat generation linked to interfacial dynamics and side reactions during cycling. While extensively explored in Li-ion and Li–S systems, its direct application to anode-less configurations remains virtually absent. For instance, thermal microcalorimetry has been successfully employed to diagnose failure mechanisms in Li–S rechargeable batteries, revealing parasitic heat signatures associated with interfacial instability [119]. Such methodological advances demonstrate the potential of *operando* calorimetry to bridge electrochemical data with thermal responses. The scarcity of direct calorimetric studies in anode-less systems represents an important gap, especially given the strong coupling between interfacial instability, lithium loss, and safety-relevant heat generation. Future work would benefit from more systematic calorimetric protocols capable of distinguishing reversible entropic contributions from irreversible heat associated with interphase growth, parasitic reactions, and contact degradation. In practical terms, benchmark measurements should ideally include heat generation as a function of current density, areal capacity, cycle number, and stack pressure, together with *postmortem* correlation to interfacial and morphological changes. Such datasets would provide a more rigorous basis for comparing the thermal stability of liquid-, hybrid, and solid-state anode-less configurations.

DSC provides quantitative insight into the thermal stability of electrolytes, interphases, and electrode–electrolyte assemblies in anode-less batteries. In sodium-based anode-less configurations, DSC has been employed to evaluate the thermal response of solid-polymer electrolytes, showing phase transitions and stability windows critical for seawater batteries [103]. Similarly, electrolyte formulations such as co-solvent engineered electrolytes for Zn anode-less systems have been investigated by DSC, demonstrating its value in mapping crystallinity, glass transition, and decomposition processes [120]. Studies on deep eutectic solvents in Zn-based systems and glass-derived solid electrolytes produced by additive manufacturing further confirm the role of DSC in characterizing thermodynamic parameters and phase behavior of unconventional electrolyte materials [121,122]. Collectively, these works highlight DSC as an indispensable tool to assess interfacial decomposition, SEI related reactions, and material stability, while also emphasizing its methodological importance in guiding the safe design of next-generation anode-less systems.

ARC is a key technique for assessing the thermal stability and runaway, as mentioned previously. By measuring self-heating rates and onset temperatures of exothermic events, ARC provides direct insights into the safety margins of both liquid- and solid-state-electrolyte systems. Although few studies have applied ARC directly to anode-less configurations, modeling approaches have highlighted the potential for catastrophic thermal events in lithium-metal all-solid-state cells, demonstrating the importance of understanding shock waves and heat propagation during thermal runaway [123]. Direct experimental support for these concerns is now emerging from ARC analyses of anode-less configurations. For instance, a 2026 study utilizing ARC and DSC conclusively showed that the electrolyte formulation and resulting SEI passivity are primary determinants of the onset temperature and violence of thermal runaway in anode-less cells, thereby translating theoretical risks into quantified thermal metrics [124]. Collectively, these studies underline ARC’s role in providing critical guidance for safe material selection, electrode architecture, and thermal management strategies in next-generation anode-less systems.

To conclude this section, Table 2 provides a concise synthesis of the characterization techniques described above, emphasizing their relevance for investigating anode-less cell architectures, the main information each method can provide, their mechanistic relevance in diagnosing key failure pathways, and illustrative examples from recent literature.

Taken together, these techniques provide complementary diagnostic windows into the dominant failure pathways of anode-less cells, linking electrochemical behavior to interfacial degradation, active lithium loss, morphological instability, void formation, and resistance evolution during cycling. Particularly valuable insight emerges when electrochemical analyses are combined with *operando* structural, chemical, or morphological probes, since such multimodal approaches allow direct correlation between cycling behavior and interphase evolution, lithium loss, void formation, or resistance growth. At the same time, techniques such as interfacial potential mapping, *operando* tomography, and thermal analyses remain comparatively underutilized in anode-less systems, despite their potential to reveal local instability, three-dimensional degradation, and heat-generation pathways that cannot be captured by electrochemical data alone.

## 3. Anode-Less with Different Electrolytes

The electrolyte plays a decisive role in the operation and reversibility of anode-less batteries, dictating not only ionic transport but also the mechanisms underlying lithium nucleation, growth, and cycling stability in the absence of a pre-deposited anode. The electrolyte’s chemical and mechanical nature govern the interfacial dynamics during lithium plating and stripping, determining parameters such as uniformity of deposition, interphase formation, and overall coulombic efficiency.

In systems employing liquid electrolytes, the high ionic conductivity and fluid nature facilitate ion transport, but often promote inhomogeneous lithium deposition and dendritic growth. In contrast, solid electrolytes, while offering improved safety and mechanical suppression of dendrites, introduce new challenges related to interfacial resistance, mechanical integrity, and chemical compatibility. Comparative studies, such as those by Lee et al. [79] and Oh et al. [55], clearly show that materials or strategies effective in liquid systems, including lithiophilic alloys and seeding layers, may exhibit markedly different behaviors in solid-state environments. In liquid-electrolyte anode-less cells, such strategies often improve plating uniformity by lowering nucleation barriers, promoting interfacial wetting, and enabling the formation of more favorable interphases. However, in solid-state systems, these same approaches do not necessarily overcome the dominant failure modes, since insufficient solid–solid contact, void formation during stripping, interfacial resistance growth, and chemomechanical mismatch may still control reversibility. Thus, a strategy that is highly effective in liquid systems may have only limited benefit in solid-state cells unless it also preserves interfacial conformity and mechanical stability throughout cycling.

Recognizing these differences is essential for establishing rational design principles. Knowledge gained from liquid-electrolyte systems can provide valuable insights into interfacial processes, but its direct translation to solid-state configurations requires careful adaptation. For this reason, this section is divided into two subsections: Section 3.1 discusses the advances and challenges in liquid electrolyte-based anode-less batteries, while Section 3.2 focuses on solid-state systems and emerging strategies toward their stable and scalable operation.

### 3.1. With Liquid Electrolytes

Anode-less battery configurations with liquid electrolytes have attracted considerable attention due to their relatively mature processing, high ionic conductivity, and compatibility with a wide range of cathode chemistries. However, the absence of an initial metal anode makes these systems particularly susceptible to interfacial instability, uncontrolled nucleation, and parasitic reactions at the current collector–electrolyte interface. As a result, significant efforts have been devoted to engineering current collectors and artificial interfaces, tailoring electrolyte formulations, and developing *in situ* plating or prelithiation strategies to improve reversibility and cycle life. This section covers recent advances in anode-less liquid-electrolyte systems, highlighting four main research directions: (i) current collector and interface engineering, (ii) electrolyte design and optimization, (iii) *in situ* strategies and prelithiation, and (iv) morphology control and advanced deposition architectures.

#### 3.1.1. Current Collector and Interface Engineering

One of the key challenges in anode-less liquid-electrolyte systems is achieving uniform Li/Na nucleation and deposition, as nonuniform plating often leads to dendritic growth and poor cycling stability. To address this, the modification of current collectors has emerged as a crucial strategy. For example, a three-dimensional copper foam coated with lithiophilic Cu_2_O and a LiF-rich interfacial layer was shown to significantly promote uniform Li deposition and enhance cycling stability [42]. Similarly, alloy-based interfacial designs have been exploited to guide sodium nucleation, as demonstrated by semi-coherent interfaces formed via alloy reactions [125] and *in situ* alloy-modified sodiophilic current collectors [126].

Besides alloying strategies, functional polymer coatings have also been investigated. For instance, a protonated polyaniline-modified copper collector enabled dendrite-free Li deposition by improving interfacial wettability and controlling nucleation [111]. In sodium systems, artificial interfacial layers based on sodium compounds have been shown to stabilize the collector–electrolyte interface and improve reversibility [127]. Additionally, interfacial engineering using two-dimensional conductive materials has emerged as an effective approach to homogenize ion flux and regulate nucleation kinetics. A holey graphene (HG) interlayer introduced onto copper collectors was shown to significantly lower the Li nucleation overpotential (from 41 to 13 mV) and reduce local current concentration, promoting uniform lithium deposition and suppressing dendritic growth. The defective and porous structure of HG also facilitates ion transport and adjusts the Fermi level of the collector (to −5.13 eV) [128], minimizing electrolyte decomposition and enabling the formation of a thin, stable SEI. As a result, anode-less Li cells with HG-modified Cu exhibited high coulombic efficiency (99.6% for 200 cycles) and smooth Li morphology even at high areal capacity (5 mAh.cm^−2^), demonstrating the effectiveness of conductive interlayers in stabilizing the Li–electrolyte interface [129]. More advanced approaches have further demonstrated the benefits of engineered sodiophilic hosts with deep depth-of-discharge capability [130] and entropy-driven interfacial stabilization through high-entropy SEI formation [81]. A self-supporting three-dimensional framework composed of nitrogen-doped hollow carbon nanofibers embedded with MgF_2_ was shown to transform *in situ* into a gradient fluorinated alloy structure during the initial plating. The resulting NaF–Mg gradient interface homogenizes the Na^+^ flux and induces outside-in directional Na deposition, enabling dendrite-free growth even at high current density (10 mA·cm^−2^) and high areal capacity (10 mAh.cm^−2^). This design demonstrates how compositional and structural gradients within the current collector can effectively couple ionic transport regulation with interfacial stability in high-rate anode-less sodium metal batteries [131]. *In situ* formation of a rapid ion diffusion interlayer based on Prussian blue analogues (PBAs) on copper current collectors has proven highly effective in enabling ultrafast Na plating/stripping. The PBA on Cu architecture exhibits a markedly lower Na^+^ diffusion barrier (~0.04 eV) and reduced nucleation overpotential compared to bare Cu, leading to homogeneous Na deposition governed by a three-dimensional instantaneous nucleation mode. Moreover, the strong interaction between the PBA interlayer and electrolyte anions promotes the formation of a NaF-rich SEI with a gradient composition that enhances ionic transport and interfacial stability. As a result, anode-less Na cells with PBAs on Cu collectors demonstrate exceptional cycling stability at 5 mA·cm^−2^ and high-rate capability up to 5 C, highlighting the critical role of interfacial ion diffusion engineering in achieving fast-charging anode-less configurations [132].

**Figure 8:**
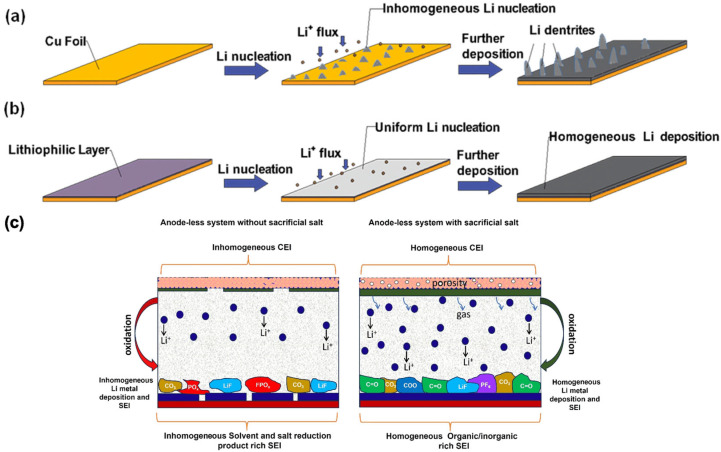
Interface engineering strategies in anode-less lithium-metal batteries: (**a**,**b**) Schematic diagrams of Li nucleation and growth on bare Cu and on Cu modified with a lithiophilic interlayer [30] (licensed under CC BY-NC 3.0); (**c**) effects of sacrificial salt interlayers (SDSs) on initial Li nucleation, redistribution, and interfacial stabilization [47] (licensed under CC BY-NC-ND 4.0).

Figure 8 illustrates current interface engineering strategies in anode-less lithium-metal batteries, including schematic representations of lithium nucleation and growth on bare copper and copper modified with a lithiophilic interlayer, as well as the effects of sacrificial salt interlayers on initial lithium nucleation, redistribution, and interfacial stabilization [30,47].

Despite the diversity of reported interface engineering strategies, their effects can be interpreted through a limited number of recurring mechanistic functions. Alloy-forming or metallophilic layers, such as Ag-, Mg-, or Sn-containing interfaces, typically reduce the nucleation barrier and interfacial energy for metal deposition, thereby increasing nucleus density and promoting more uniform plating. In contrast, fluorinated, inorganic, or polymer-based coatings can regulate ion flux and modify interphase chemistry, often suppressing parasitic reactions while promoting more stable and mechanically robust interphases. Crystallographic or structural control of the current collector, including oriented surfaces and porous or conductive interlayers, further contributes by homogenizing local current distribution and guiding deposition morphology. In practice, the best-performing interfaces usually combine these effects such that nucleation control, flux homogenization, and interphase stabilization act synergistically rather than independently.

From a practical perspective, lithiophilic seed layers and interfacial coatings should not be evaluated solely on their ability to reduce nucleation barriers or improve cycling behavior. Their real viability also depends on the associated mass and thickness penalty, the amount of inactive material introduced, and the compatibility of the modification route with scalable cell manufacturing. In this context, ultrathin and conformal coatings are generally more attractive than thick or structurally complex interlayers, since excessive added mass or processing complexity may offset the energy-density advantage of anode-less architectures. Therefore, future studies should more systematically report coating thickness, areal loading, and process compatibility to assess the practical benefit of interfacial engineering strategies.

#### 3.1.2. Electrolyte Design and Optimization

Electrolyte formulation plays a pivotal role in determining the performance of anode-less liquid-electrolyte systems, as it governs ion transport, interphase formation, and overall interfacial stability. Electrolyte composition regulates the solvation structure of the plating ion and thus the sequence of interfacial reactions that occur during metal deposition. Solvent polarity, salt concentration, and additive chemistry determine the fraction of free solvent, the degree of anion coordination, and the desolvation barrier at the current collector surface, thereby influencing whether the interphase is predominantly solvent-derived or anion-derived [19,20,133]. This in turn affects interphase composition, mechanical robustness, ionic transport, and resistance evolution. In anode-less architectures, these effects are especially critical, because the absence of an excess metal reservoir makes the system highly sensitive to even small losses arising from parasitic interfacial reactions or nonuniform plating [18,19]. A detailed experimental study introduced strategic electrolyte design principles for Li metal-free systems, emphasizing solvent polarity, salt concentration, and additive chemistry to tailor interfacial features and suppress parasitic reactions [133]. Hybrid solvent systems, such as carbonate glyme blends, have been developed to balance ionic conductivity with chemical stability, resulting in improved Li deposition behavior [134]. Another dominant approach has been the use of high-concentration or dual-salt electrolytes, which promote the formation of robust interphases and suppress dendrite growth [135,136,137].

Systematic high-throughput studies have further expanded our understanding of electrolyte effects. A comparative evaluation of 65 electrolyte formulations in anode-less NMC811 (LiNi_0.8_Mn_0.1_Co_0.1_O_2_) pouch cells provided critical insights into electrolyte–electrode interactions and failure mechanisms [138]. Beyond conventional solvents and salts, ionic liquid electrolytes have also been applied, including superconcentrated phosphonium-based systems [139].

More recent work has advanced the concept of “beyond concentrated” electrolytes for sodium systems [140] and entropy-driven electrolytes with extremely high salt contents for lithium [141]. In parallel, the extension of electrolyte optimization principles to other alkali-metal systems has led to significant breakthroughs. An integrated dual-ion strategy employing a low-temperature electrolyte based on DME/G2 and KTFSI enabled reversible potassium plating/stripping even at −40 °C. The carefully balanced solvation structure reduced the desolvation energy of K^+^ ions and stabilized the interphase by forming a KF- and K_2_S-rich SEI, resulting in high coulombic efficiency and excellent rate performance. This electrolyte design also delivered a record energy density of 407 Wh·kg^−1^, demonstrating that rational solvent–salt coordination and anion engineering can effectively extend the anode-less concept to potassium systems under cryogenic conditions [142]. In addition, *operando* studies have revealed failure origins in anode-less systems, highlighting the critical role of electrolyte decomposition and morphological evolution [143].

Collectively, these advances demonstrate that electrolyte design is a central determinant of long-term stability in anode-less cells.

#### 3.1.3. *In Situ* Strategies and Nucleation

Beyond passive stabilization approaches, *in situ* plating strategies and prelithiation agents have been developed to compensate for irreversible capacity loss and facilitate the initial formation of a metallic anode. In sodium systems, direct *in situ* plating of Na metal on the current collector was demonstrated as a feasible route to achieving rechargeable anode-less operation [144]. Similarly, rechargeable anode-less (Li-metal) batteries have been realized by enabling controlled *in situ* deposition of Li during the initial cycles [23].

Complementary to these strategies, sacrificial salts added to the electrolyte have been used to provide extra lithium and improve the coulombic efficiency of the initial cycles. *Operando* optical microscopy revealed critical insights into the growth of dead Li in such systems [47]. In parallel, solid prelithiation agents such as lithium carbide (Li_2_C_2_) have been proposed as controlled sources of Li, effectively enhancing both the energy density and cycling stability of anode-less cells [145].

In addition to chemical and material-based approaches, the electrochemical formation protocol itself has been shown to strongly influence the reversibility of Li plating in anode-less configurations. It was demonstrated that a slower formation rate during the initial cycles (e.g., 0.05 mA·cm^−2^) promotes uniform Li nucleation, resulting in a dense and compact deposition layer and a stable, LiF-rich SEI. Conversely, higher formation rates (≥0.5 mA·cm^−2^) lead to nonuniform nucleation, porous Li morphology, and increased electrolyte decomposition, ultimately accelerating capacity fade. These findings highlight that tuning the initial plating kinetics is an effective *in situ* strategy to control nucleation dynamics and enhance the long-term cycling stability of anode-less lithium-metal batteries [146].

Beyond electrochemical parameters, mechanical factors have also been shown to play a decisive role in the evolution of Li deposition and inactive lithium formation. Using *in situ* nuclear magnetic resonance (NMR), it was revealed that stacking pressure directly influences Li nucleation behavior and the generation of electrically isolated “dead” Li in anode-less cells. Excessive external pressure promotes heterogeneous plating and accelerates the accumulation of inactive metallic Li, while moderate pressure maintains interfacial contact and suppresses void formation during cycling. These findings establish an optimal pressure window that minimizes dead-Li buildup and improves coulombic efficiency, offering new mechanistic insights into the coupling of mechanical and electrochemical processes in anode-less configurations [147].

Extending these principles beyond lithium and sodium systems, anode-less potassium metal batteries have also been demonstrated under optimized *in situ* plating conditions. Low-temperature operation (down to −40 °C) was achieved with a dilute KPF_6_–DME electrolyte combined with a polydimethylsiloxane (PDMS) additive, which facilitated the formation of a robust hybrid SEI composed of inorganic KF and polymeric Si–O–K species. This interphase architecture effectively suppressed dendritic growth and ensured uniform K deposition, enabling high coulombic efficiency (~99.8%) and an energy density of 152 Wh·kg^−1^. Such results highlight the adaptability of *in situ* formation and interfacial engineering strategies to other alkali-metal anode-less systems, even under extreme operating conditions [148].

These strategies represent a promising complementary pathway to overcome the intrinsic lithium/sodium deficiency of anode-less configurations.

#### 3.1.4. Advanced Morphologies and Deposition Control

The final critical aspect in anode-less liquid-electrolyte systems is the control of metal deposition morphology. Ideally, Li/Na should be deposited as dense, uniform layers with minimal porosity, thereby reducing the risk of dendritic growth and improving cycling efficiency. A key demonstration in this field achieved highly compact and homogeneous Li deposits, enabling high efficiency and long-life cycling in anode-less lithium-metal batteries [149].

In sodium systems, the design of hierarchical three-dimensional hosts with enhanced sodiophilicity were shown to significantly promote uniform Na deposition and long-term stability [150]. Together, these results highlight the importance of morphological control in stabilizing anode-less architectures, particularly under practical high-capacity and high-current-density conditions.

To provide a concise overview of the experimental work on anode-less cells with liquid electrolytes, Table 3 summarizes key studies in chronological order, highlighting cell configurations, electrolyte composition, interfacial modifications, and principal electrochemical performance metrics. This compilation allows for an immediate comparison of strategies and results, facilitating the identification of trends and advances in electrolyte design and interface engineering.

Table 3 shows that no single electrolyte-design strategy can yet be considered universally optimal for anode-less liquid-electrolyte systems. Instead, the most successful reports generally combine electrolyte optimization with interface engineering and deposition control. High-concentration, dual-salt, and solvation structure-engineered electrolytes often improve cycling stability by reducing free-solvent reactivity and promoting more robust interphases, but their apparent benefits remain strongly system-dependent and must be interpreted together with cathode chemistry, current density, areal capacity, and formation protocol. A broader trend that emerges is that high coulombic efficiency alone does not guarantee a long cycle life unless it is accompanied by stable interfacial resistance, low parasitic reactivity, and controlled deposition morphology. In this sense, the data in Table 3 suggest that electrolyte formulation should not be viewed as an isolated variable, but rather as one component of an integrated interfacial design strategy.

Electrolyte formulation plays a pivotal role in determining the performance of anode-less liquid-electrolyte systems, as it governs ion transport, interphase formation, and overall interfacial stability. Electrolyte composition regulates the solvation structure of the plating ion and thus the sequence of interfacial reactions that occur during metal deposition. Solvent polarity, salt concentration, and additive chemistry determine the fraction of free solvent, the degree of anion coordination, and the desolvation barrier at the current collector surface, thereby influencing whether the interphase is predominantly solvent-derived or anion-derived [133,135,136,137]. This in turn affects interphase composition, mechanical robustness, ionic transport, and resistance evolution. In anode-less architectures, these effects are especially critical because the absence of an excess metal reservoir makes the system highly sensitive to even small losses arising from parasitic interfacial reactions or nonuniform plating [133,134].

Coulombic efficiency (CE) should also be interpreted quantitatively in anode-less systems, since even small irreversible losses accumulate rapidly in the absence of an excess metal reservoir. Under the idealized approximation that the remaining fraction of cyclable lithium after N cycles is given by ${\;CE}^{N},$ and assuming 80% retention of the initial cyclable lithium, the cycle number can be estimated from $N=\frac{ln(0.8)}{\ln\left(CE\right)}$, where CE is expressed as a decimal fraction and *N* is the number of cycles. This shows that 99.0%, 99.5%, and 99.9% CE would correspond to approximately 22, 45, and 223 cycles, respectively, whereas achieving 300 cycles at 80% retention would require a CE of about 99.93%. We also note that this is an idealized estimate and that practical service life additionally depends on morphology evolution, interfacial resistance growth, and the specific testing conditions [81,129,142]. In practice, the significance of CE depends strongly on the testing conditions, including areal capacity, current density, cathode loading, formation protocol, and interfacial stability. Thus, high CE is a necessary, but not sufficient condition for long service life unless accompanied by stable morphology, low parasitic reactivity, and controlled resistance growth [129,138,146].

Although some recent studies have reported extended cycling performance, and in selected cases, more than 300 cycles with relatively high CE, such results should not yet be taken as definitive proof of universally stable interphase formation under fully practical conditions [81,129,142]. In many cases, long cycle life is demonstrated only under specific combinations of areal capacity, cathode loading, electrolyte amount, current density, or formation protocol, and direct evidence of a chemically and mechanically stable SEI over extended cycling remains comparatively limited [129,142]. Therefore, current long-term data are better interpreted as promising indications of improved interfacial stabilization rather than conclusive validation that SEI related degradation has been fully resolved in practical anode-less cells.

Performance claims in anode-less systems should also be interpreted with caution unless the corresponding testing conditions are clearly specified. Metrics such as cycling stability or CE can appear highly favorable under one set of conditions, but may not translate directly to practical relevance if areal capacity, cathode loading, current density, electrolyte amount, stack pressure, or lithium inventory constraints are substantially different. For this reason, comparisons across studies should be made only in the context of the full cell design and testing protocol, rather than because of isolated performance indicators alone.

As liquid electrolyte-based anode-less systems advance, the inherent limitations of liquid media, such as parasitic reactions, limited electrochemical stability windows, and safety concerns, motivate the shift toward solid or quasi-solid systems. Recent studies have begun to bridge this divide by exploring *in situ* transformations or hybrid systems that combine liquid, gel, and solid features. For instance, careful selection of solvent and additive has been shown to enable the conversion of a liquid electrolyte into a gel–solid polymer electrolyte [151], thereby stabilizing both conventional and anode-less assemblies. Similarly, the incorporation of liquid metals within an *in situ* polymerized electrolyte demonstrated a self-healing mechanism through reversible liquid–solid–liquid transitions [152]. In addition, tuning the salt concentration in gel–solid polymer electrolytes was shown to enhance ionic conduction and stabilize high voltage cathodes [153]. A broader perspective is provided by studies that systematically investigate strategies applicable to both liquid and solid electrolytes, such as the use of lithiophilic seeds to guide uniform metal nucleation and improve reversibility [55]. Collectively, these studies illustrate viable strategies to combine the advantages of liquid ionic mobility with the stability and safety associated with solid-like electrolytes. Although hybrid systems may serve as useful developmental bridges, they should not be interpreted as direct proxies for fully dry solid-state cells, because residual liquid or gel phases can substantially alter interfacial wetting, contact retention, and apparent electrochemical reversibility. The next section discusses the state of the art in solid electrolyte anode-less systems, their challenges, and future directions.

To better distinguish the governing constraints of liquid- and solid-state anode-less batteries, a comparative synthesis is provided in Table 4. Although both classes of systems aim to enable reversible metal plating/stripping without a host anode, their dominant failure modes, design variables, and practical trade-offs differ substantially.

These distinctions help explain why strategies that improve reversibility in liquid-electrolyte systems cannot always be translated directly to solid-state configurations, where interfacial contact retention, pressure, and chemomechanical stability become dominant design constraints.

### 3.2. With Solid Electrolytes

In anode-less solid-state batteries, cell build and stack pressure are not merely assembly parameters, but critical design variables that directly influence interfacial contact, current distribution, and the reversibility of metal plating/stripping. Because the negative electrode is formed *in situ* on a bare current collector, insufficient pressure or poor cell-level mechanical compliance can promote interfacial gaps, nonuniform deposition, local current constriction, void formation during stripping, and progressive resistance growth [154]. Conversely, although applied pressure can improve contact retention and cycling stability, excessive pressure may introduce additional mechanical constraints and reduce the practical viability of the system at the stack level. For this reason, cell architecture, pressure management, and mechanical integration must be considered together with electrolyte and interface design when assessing the performance of anode-less solid-state cells [64,155].

In anode-less batteries employing solid electrolytes, the electrochemical processes of lithium plating and stripping are intrinsically linked to the mechanical and chemical nature of the solid interfaces. Recent perspectives have highlighted that the behavior of all-solid-state systems cannot be understood solely from an electrochemical viewpoint. The work in ref. [64] emphasizes that the coupling between ionic transport, interfacial reactions, and mechanical stress governs the reversibility and morphological evolution of lithium. Local stresses and interfacial deformations arising during cycling can alter ion pathways and reaction kinetics, ultimately determining the long-term stability of the system.

Complementarily, the perspective in ref. [95] provides a comprehensive overview of recently explored material and design strategies to improve the performance of anode-less solid-state configurations. It categorizes progress according to electrolyte type, highlights challenges such as maintaining interfacial contact and optimizing stack pressure, and outlines future directions involving interlayer engineering, lithiophilic scaffolds, and mechanically adaptive electrolytes.

Within this framework, ongoing research focuses on clarifying how the intrinsic properties of solid electrolytes, such as elasticity, plasticity, and fracture resistance, influence lithium nucleation, interface evolution, and cycling stability. Understanding these interdependent electro/chemo/mechanical processes is essential for guiding the rational development of next-generation anode-less all-solid-state battery architectures.

#### 3.2.1. Fundamentals of Anode-Less Operation in Solid-State Cells

The reversibility of lithium deposition and stripping in anode-less solid-state batteries is governed by the interplay among electrochemical kinetics, interfacial chemistry, and mechanically induced deformation. Unlike liquid-based systems, which allow rapid interfacial rearrangement and local accommodation, solid electrolytes impose structural rigidity and mechanical constraints that intimately link ionic transport with stress generation. This electro-chemomechanical coupling dictates how lithium nucleates, grows, and redistributes during cycling.

Studies on glassy and amorphous alkali-ion solid electrolytes demonstrated that deformable matrices can relax interfacial stress and suppress filamentary growth [24]. These observations provided some of the first experimental evidence that mechanical compliance and ionic conduction are interdependent in solid-state environments. Building on this conceptual framework, later investigations on practical anode-less configurations revealed that controlled conditioning steps and interfacial relaxation phenomena can substantially improve lithium reversibility and long-term cycling stability [26].

Historical developments include “lithium-free” thin-film batteries that utilize *in situ* plating of the lithium anode from cathode lithium sources through thin lithium phosphorus oxynitride (LiPON) electrolytes, demonstrating the foundational principle of anode-less operation with deposited lithium-metal anodes formed dynamically during cycling in a solid-state device architecture. This early thin-film configuration, despite its simplified planar geometry and ceramic electrolyte, introduced essential concepts of *in situ* anode formation, plating efficiency, and interphase control that remain highly relevant to modern solid-state anode-less cells [156].

Pushing the concept of anode-less operation to its theoretical limit, recent work has demonstrated that persistent battery-like discharge can be achieved in a truly “electrodeless” or “capacity-less” solid-state architecture. In a system employing a ferroelectric Na_2.99_Ba_0.005_OCl electrolytes with inert Inconel 625 and aluminum current collectors, a stable discharge plateau at ~1.1 V was maintained for months without any conventional battery-like oxidation/reduction reactions [157]. This paradigm-shifting example demonstrates that the fundamental driving force, the difference in electron chemical potential between the current collectors, can sustain energy delivery through purely interfacial electrostatic mechanisms such as electrical double-layer formation. This underscores that the solid electrolyte is not a passive ionic conductor, but an active component that defines the thermodynamic and kinetic landscape for energy storage, even in the absence of conventional active materials [157].

*Operando* and modeling studies further clarified the role of mechanical feedback. Localized stress accumulation was correlated with nonuniform current distribution and filament initiation, even under nominally homogeneous conditions [158]. Poor interfacial adhesion and void formation were found to accelerate delamination and contact loss, compromising electrochemical reversibility [159].

Thermomechanical factors also play a decisive role. Temperature-dependent transitions from dense to filamentary morphologies demonstrate that both lithium mobility and solid-electrolyte relaxation contribute to interfacial evolution [160]. Stress concentrations can promote electronic leakage and catastrophic failure, strongly linking electrochemical instability with mechanical degradation pathways [161].

Advanced *operando* characterization has shown that transient reaction layers and anisotropic ion-transport pathways strongly influence the boundary between reversible and irreversible cycling [11,162]. Collectively, these findings converge on the notion that achieving stable lithium cycling in anode-less solid-state cells requires an integrated understanding of electrochemistry, mechanics, and interfacial materials physics. The solid electrolyte cannot be regarded as a passive ion conductor: instead, it actively shapes nucleation dynamics, stress distribution, and chemical stability at the Li–electrolyte interface.

Work on anode-less solid-state polymer cells has reinforced this fundamental picture by quantitatively separating lithium loss into solid-electrolyte interphase (SEI) formation and electrically isolated “dead” Li through *operando* solid-state NMR and complementary microscopy/spectroscopy. In a LiPF_6_-based PDOL electrolyte, the initial capacity loss is largely SEI-dominated, while subsequent degradation is governed by nearly linear dead-Li accumulation, and reformulating the electrolyte with LiFSI and Cs^+^ additives to promote inorganic-rich, mechanically robust SEI, and smoother Li plating/stripping shifts the system toward an SEI-dominated regime with strongly suppressed dead-Li growth and significantly higher coulombic efficiencies in anode-less solid-state architectures [163].

Building on these principles, the following section examines how major classes of solid electrolytes—halides, oxides, sulfides, polymers, and hybrid systems—govern interfacial behavior and overall cycling reversibility in anode-less solid-state batteries.

#### 3.2.2. Influence of Electrolyte Family and Chemistry

The electrolyte chemistry strongly influences lithium deposition and stripping in anode-less solid-state batteries, acting simultaneously as an ionic conductor, mechanical buffer, and chemically active medium. Its intrinsic properties—ionic transport, electrochemical stability, mechanical compliance, and interfacial reactivity—dictate lithium nucleation dynamics, morphology evolution, and cycle life. This section examines the behavior of different classes of solid electrolytes—inorganic (halides, oxides, sulfides), solid polymer electrolytes, and hybrid/composite systems—highlighting insights from recent experimental and *operando* studies.

##### Inorganic Solid Electrolytes

**Halides and Oxyhalides:** Halide electrolytes have recently emerged as a promising platform for anode-less designs due to their intrinsic chemical stability against lithium and moderate elastic modulus, which allows for self-adaptive interfacial contact during cycling.

Systems such as Li_3_YCl_6_ and Li_3_InCl_6_ have demonstrated homogeneous Li nucleation and improved reversibility under limited stack pressure conditions [164].

The introduction of Ba doping into the Li_3_OCl lattice, forming Li_2.99_Ba_0.005_ClO, further exemplifies how compositional engineering can tune both ionic transport and mechanical deformability to favor stress relaxation and uniform Li plating [26].

*In operando* investigations have confirmed that local mechanical relaxation plays a decisive role in suppressing stress driven filament growth during early nucleation [158].

In addition to chemical and mechanical advantages, halide electrolytes exhibit wide electrochemical windows and excellent compatibility with high voltage cathodes, making them suitable for energy dense anode-less configurations. Oxyhalide derivatives with partial oxygen substitution further enhance interface robustness by introducing mixed ionic–electronic buffering effects, reducing Li depletion at the current collector.

**Oxides and Garnets:** Oxide-based solid electrolytes, especially garnet-type Li_7_La_3_Zr_2_O_12_ (LLZO), combine high ionic conductivity with excellent stability against both Li metal and cathode materials. However, their high elastic moduli and poor deformability lead to contact loss during stripping. Composite strategies, such as laminating ultrathin LLZTO-based layers on the current collector, enable more uniform plating and stripping behavior [165].

Thin-film oxides such as LiPON, many used in microbatteries, provide an instructive model for understanding interfacial dynamics under zero excess Li. Observations of Li plating at Ti–LiPON and LiPON–LiPON artificial interfaces have elucidated the role of charge density and local electric field gradients in guiding Li growth morphology [166,167].

Oxide films with reversed structural configurations, such as SS–Li–LiPON–Li_x_V_2_O_5_–Cu, demonstrate how architecture and interfacial polarity control Li redistribution and reversibility [168]. These findings underscore that in rigid oxides, achieving uniform plating requires balancing electronic insulation with localized strain accommodation, a recurring theme across solid electrolytes.

**Sulfides and Argyrodites:** Sulfide-based electrolytes, including Li_6_PS_5_Cl, Li_6_PS_5_Br, and related argyrodites, are widely recognized for their high ionic conductivity (>10^−3^ S·cm^−1^) and low shear modulus, enabling intimate solid–solid contact and facile Li transport. However, their narrow electrochemical window and reactivity with Li lead to interphase formation and void evolution.

*Operando* spectroscopy and electron microscopy studies have shown that interfacial decomposition and uneven Li plating evolve dynamically during cycling [162].

Chemical modification strategies, such as Ag exsolution from Li-argyrodite matrices, have demonstrated self-formed lithiophilic sites that stabilize the Li interface and reduce impedance growth [169].

Similarly, In-doped Ag coatings or dual metal interlayers act as kinetic regulators, homogenizing Li flux and preventing dendrite propagation [58,170].

Further insight into degradation mechanisms has been obtained through aging and failure studies, which reveal that filament-induced breakdown and interfacial delamination are closely tied to chemomechanical coupling [161,171].

##### Solid Polymer Electrolytes

Solid polymer electrolytes provide mechanical flexibility and good interface wettability, but suffer from limited ionic conductivity at room temperature. Studies on PEO-based solid polymer electrolytes have shown that polymer segmental motion governs Li-ion migration and that optimizing interfacial chemistry is essential to minimize irreversible Li loss [172].

Surface modification of Cu current collectors with protonated polyaniline and other conductive polymers improves lithiophilicity and suppresses local current hotspots, leading to smoother Li deposition [111].

Likewise, hybrid polymer networks within *situ* polymerized liquid–metal components or rapid ion diffusion interlayers have demonstrated significantly enhanced Li uniformity and rate capability [152].

In anode-less solid polymer cells, dedicated stripping/plating studies have further shown that the electrolyte formulation and cycling protocol critically determine the fraction of lithium that can be reversibly recovered from the current collector, with tailored polymer electrolytes enabling markedly higher stripping efficiencies and revealing the fundamental limits of reversibility in polymer-based anode-less architectures [173].

##### Hybrid and Solid Composite Electrolytes

In anode-less solid-state batteries, the electrolyte must meet a unique set of concurrent requirements: high ionic conductivity, negligible electronic conductivity, chemical and mechanical robustness, and interfacial compatibility with the *in situ* plated metal. Meeting these concurrent requirements contribute to the exploration of hybrid and composite solid electrolytes that integrate ceramic and polymeric phases to combine the advantages of both. Ceramic components provide mechanical strength and suppress dendrite penetration, while the polymeric phase improves flexibility, interfacial contact, and processability.

Hybrid polymer–ceramic systems have shown remarkable potential in lithium-based solid-state batteries, where polymer infiltration into oxide frameworks or the incorporation of ferroelectric fillers improves both ionic transport and interface stability. A representative example is the use of a PVDF-based hybrid solid electrolyte containing ferroelectric oxide fillers that enhance conductivity and suppress dendritic growth, contributing to longer cycling stability and more uniform lithium deposition [174]. Although this work was not strictly anode-less, the electrolyte design principles demonstrated are directly transferable to anode-less configurations, where uniform nucleation and smooth *in situ* metal growth are essential for achieving high coulombic efficiency.

A comprehensive overview of these strategies and their technological implications is presented in a recent review discussing the key challenges of anode-less solid-state sodium batteries, particularly the interfacial instability and dendrite formation that limit cycle life and efficiency. This work highlights hybrid and composite electrolytes as promising routes to overcome these bottlenecks by uniting mechanical rigidity, ionic conductivity, and adaptive interfacial chemistry within a single architecture [13]. Collectively, these findings emphasize that the synergy between hybrid electrolyte design and anode-less configurations offers a promising pathway for achieving high-energy-density and durable solid-state batteries.

Taken together, electrolyte chemistry dictates not only the electrochemical and mechanical dynamics of lithium plating but also the architectural design required for stability. The next Section 3.2.3 will explore how interface and interlayer engineering, through lithiophilic coatings, metallic buffers, and mechanically adaptive layers, further refines these electrolyte-dependent processes.

#### 3.2.3. Interface and Interlayer Engineering

Interfacial and interlayer engineering plays a central role in enabling stable cycling of anode-less solid-state batteries. The chemical, mechanical, and electrochemical dynamics at the current collector–electrolyte interface critically determine lithium, sodium, or potassium nucleation behavior and long-term reversibility. This subsection provides a concise overview of recent advances in interface design, including the use of metal or alloy buffer layers, composite and artificial interphases, and surface modification of current collectors. The discussion integrates findings from both *ex situ* and *operando* studies to elucidate the mechanisms underlying uniform metal deposition and interfacial stabilization across different solid electrolyte chemistries.

##### Fundamental Interfacial Instabilities and Design Principles

In anode-less solid-state batteries, the interface between the deposited alkali metal and the solid electrolyte is intrinsically unstable due to a combination of chemical reactivity, mechanical mismatch, and uneven current distribution. These factors can lead to void formation, localized dendritic growth, and rapid capacity loss, particularly during repeated plating and stripping cycles. The adhesion between the current collector and the deposited metal, as well as the mechanical compliance of the solid electrolyte, plays a crucial role in determining the uniformity of metal nucleation and growth [159]. *In operando* studies have revealed that interphase dynamics are highly sensitive to both the chemical composition of the solid electrolyte and the applied stack pressure, highlighting the need for strategies that simultaneously address chemical stability and mechanical integrity [161,162]. Chemical inertness of the current collector itself is a critical, yet often overlooked design criterion. In systems employing highly reactive solid electrolytes (e.g., sulfides, ferroelectrics), the collector must resist corrosion and parasitic reactions over extended cycling. For instance, Inconel 625 (a nickel-based superalloy) has demonstrated exceptional interfacial stability in contact with a Na-based ferroelectric electrolyte over one year of operation, with no detectable chemical degradation via *postmortem* SEM–EDX analysis [157]. Across lithium, sodium, and potassium systems, these principles remain broadly applicable: the modulation of surface energy, interface adhesion, and ion flux directionality are central to achieving homogeneous metal deposition and minimizing early-stage failure.

##### Metal-Based and Lithiophilic Interlayers

The incorporation of metal-based interlayers has proven to be one of the most effective strategies to control nucleation and growth of alkali metals in anode-less solid-state batteries. Such interlayers, typically composed of lithiophilic, sodiophilic, or potassiophilic metals, serve to reduce nucleation overpotentials and guide uniform metal deposition. For instance, Ag/C buffer layers have been shown to facilitate homogeneous lithium plating and stripping through reversible alloying and localized electron redistribution [175]. Similarly, kinetically engineered dual metal layers, designed with complementary lithiophilicity, suppress dendrite formation while enhancing plating efficiency and cycle life [58]. Modifications such as In doping of Ag coatings have further demonstrated the ability to stabilize the interface by improving adhesion and reducing interfacial resistance [170]. Beyond lithium, exsolution phenomena in silver-containing argyrodite electrolytes provide an intrinsic mechanism for self-generated metallic interlayers, suggesting that similar strategies could be extended to sodium and potassium systems [169]. Advanced characterization techniques such as scanning Kelvin probe (SKP) are instrumental in the rational selection and validation of these interlayers. By directly mapping the surface potential (work function) of candidate materials, SKP enables the quantitative prediction of interfacial electrochemical potentials. This approach was decisively used to select Inconel 625 as a positive current collector in a Na-based solid-state cell, where its measured chemical potential ensured a suitable voltage difference with the aluminum counter-electrode [157]. Collectively, these studies highlight that careful selection and engineering of metal interlayers is a versatile and broadly applicable approach to enhance uniformity, suppress dendrites, and improve the electrochemical stability of anode-less cells across different alkali metals.

The metallic lithium anode formed *in situ* on a copper current collector modified with a lithiophilic metal interlayer creates a stable interface with the solid electrolyte, promoting uniform lithium deposition and enabling high coulombic efficiency and long cycle life in “lithium-free” solid-state batteries [176].

##### Composite and Artificial Interlayers

Beyond single metal layers, composite and artificial interlayers offer additional chemical and mechanical functionalities that are essential for stabilizing alkali-metal deposition in anode-less solid-state batteries. Composite interlayers, combining metals with carbonaceous materials or ceramic particles, can enhance electronic conductivity while simultaneously providing mechanical compliance to accommodate volume changes during plating and stripping [101]. Artificial solid-electrolyte interphases formed via sacrificial thin films, such as MoS_2_, have been employed to create a chemically stable, ion-conductive barrier that mitigates direct contact between the reactive metal and the solid electrolyte [177]. These layers can be designed to selectively allow ion transport while preventing electron leakage, thereby suppressing dendrite nucleation and propagation. Conditioning and the introduction of additional interfacial layers, such as ZnO deposition, are crucial for establishing a stable interface between the deposited metal and the solid electrolyte, promoting homogeneous lithium nucleation and improving the electrochemical performance of anode-less cells [26]. By integrating composite and nucleation interlayers, researchers have demonstrated more uniform metal deposition, lower interfacial polarization, and improved capacity retention over repeated cycling across lithium, sodium, and potassium systems, underlining the general applicability of this approach in diverse alkali-metal anode-less configurations.

##### Collector Surface Modification and Morphology Control

Modifying the surface of current collectors represents a complementary strategy to interlayers for controlling alkali-metal deposition in anode-less solid-state batteries. Surface texturing and microstructuring can enhance the effective surface area, modulate local electric fields, and direct ion flux, thereby promoting uniform nucleation and mitigating void formation. For example, microstructured copper electrodes have been shown to improve sodium deposition by increasing contact area and reducing interfacial resistance, demonstrating that morphological control is an effective tool even beyond lithium-based systems [77]. Additive manufacturing (AM) techniques, such as metal fused filament fabrication (FFF), offer unprecedented design freedom for creating collectors with optimized, complex geometries that maximize interfacial contact with solid electrolytes. This approach was successfully employed to fabricate a 3D Inconel 625 component, which, combined with design for additive manufacturing (DFAM) principles, ensured intimate electrolyte infiltration and robust mechanical integration in a long-life solid-state cell [157]. Collectively, these approaches highlight that careful design of collector morphology and surface chemistry can synergize with interlayers to stabilize the alkali metal–electrolyte interface across Li, Na, and K systems.

##### Extension of Interfacial Design Principles Across Alkali-Metal Systems (Li, Na, K)

Although Li-based systems remain the primary focus of this review, brief discussion of Na- and K-based systems is included here as a comparative extension of the anode-less concept. This subsection is not intended to provide the same level of depth for all alkali-metal chemistries, but rather to highlight how key interfacial phenomena, deposition behavior, and design challenges may evolve beyond lithium. Most interfacial strategies have been initially developed for lithium-based anode-less solid-state batteries, but the underlying principles are broadly applicable across sodium and potassium systems. The fundamental requirements (uniform nucleation, mechanical compliance, and stable chemical contact) remain consistent, even as the alkali-metal properties differ in terms of ionic radius, redox potential, and surface energy. For instance, surface modification of copper collectors has been shown to control sodium deposition similarly to lithium, with microstructuring enhancing contact area and directing ion flux [77]. The spontaneous polarization of the ferroelectric electrolyte generates dipoles at the Cu–Na_3_ClO interface, modulating metallic sodium deposition and enhancing interface stability in solid-state sodium batteries [66]. In sodium systems, the spontaneous polarization of ferroelectric electrolytes such as Na_3_ClO, with relative permittivity reaching up to 10^13^ in some cases, generates dipoles at interfaces that modulate metallic sodium deposition and contribute to interface stabilization. These ferroelectric properties present a universal mechanism to mitigate dendrite formation and enhance cycling stability, applicable across Na and K anode-less solid-state technologies. Furthermore, the use of cellulose membranes as dielectric separators interacts favorably with alkali-metal ions, promoting efficient ionic conduction and polarization phenomena relevant to interlayer design [29]. The exploration of extreme architectures, such as “electrodeless” cells, further validates the universality of interfacial principles. In a sodium-based system, the sustained discharge from purely electrostatic double layers at inert metal–ferroelectric electrolyte interfaces demonstrates that the core challenge (managing the potential difference and charge distribution at the interface) is a unifying theme across all alkali-metal and even non-faradaic solid-state batteries [157]. These examples highlight that careful engineering of interlayers, current collector surfaces, and deposition frameworks provides a universal toolbox to mitigate dendrite formation, enhance cycling stability, and optimize electrochemical performance in solid-state ALMB regardless of the alkali-metal chemistry.

#### 3.2.4. Characterization and *in Operando* and *Postmortem* Studies in Solid ALMB

Advanced characterization techniques are essential for understanding the dynamic processes at the metal–electrolyte interface in anode-less solid-state batteries. *In operando* approaches provide real-time insight into nucleation, growth, interphase evolution, and mechanical effects during cycling, while *postmortem* analyses reveal the final chemical and morphological state of the interface.

Three-dimensional *in operando* microscopy and *in situ* NMR have been particularly effective in visualizing lithium, sodium, or potassium deposition and linking interfacial behavior to electrochemical performance. *In operando* 3D imaging directly captures filament formation, dendritic growth, and local stress evolution, enabling mechanistic correlations between plating conditions and failure pathways [158]. *In operando* optical microscopy in anode-less configurations has enabled direct plan-view visualization of lithium nucleation, dendrite propagation, and dead-lithium accumulation at transparent current collectors, providing a complementary, real-time morphological probe of interfacial degradation in practical cells [178].

In addition to microscopy and spectroscopy, quantitative analysis of inactive lithium can be significantly strengthened by combining NMR with gas analysis-based methods. *Operando* or *ex situ* solid-state NMR is particularly valuable for separating capacity loss associated with interphase formation from the accumulation of electrically isolated “dead” lithium, thereby providing a more mechanistic view of lithium inventory decay [179,180]. Complementary titration- or gas evolution-based methods, such as titration gas chromatography or related mass-spectrometric approaches, can further help quantify inactive lithium and identify parasitic decomposition pathways, especially when multiple chemically distinct inactive species may coexist [179,181]. Used together, these techniques improve the interpretation of coulombic inefficiency by distinguishing whether lithium is consumed mainly by interphase growth, trapped as dead lithium, or diverted into secondary chemical reactions [179,180,181].

Complementary *postmortem* analyses, such as laser-induced breakdown spectroscopy (LIBS), provide detailed compositional maps of the interphase after cycling. These measurements help quantify degradation products, assess interlayer effectiveness, and identify chemical heterogeneities that may contribute to capacity fade [31]. In systems dominated by electric double-layer (EDL) capacitance, such as in “capacity-less” solid-state architectures, characterization must probe interfacial electrostatics and chemical potential directly. In such a cell with a ferroelectric Na_2.99_Ba_0.005_OCl electrolyte, *postmortem* scanning Kelvin probe (SKP) mapping was crucial to confirm the exceptional stability of the Inconel 625 current collector. SKP measurements showed that its surface potential (and thus chemical potential) remained virtually unchanged after one year of continuous operation, providing direct evidence of electrochemical inertness and interfacial robustness [157]. This highlights SKP as a powerful tool for validating the long-term stability of current collectors in aggressive solid-state environments [157].

Although most studies have focused on lithium systems, *operando* and *postmortem* techniques have been successfully applied to sodium and potassium anode-less cells. For example, microstructured copper collectors and gradient frameworks in sodium cells reveal analogous interfacial dynamics, including directional growth and effective interlayer stabilization [77,131].

First-principle simulations complement experimental characterization by providing atomic scale understanding of interface energetics. Density functional theory (DFT) calculations of the work function, convertible in chemical potentials, for various Inconel 625 surfaces provided a theoretical basis for the measured chemical potential and predicted the open-circuit voltage of an Al–Inconel cell with remarkable accuracy, demonstrating how computational modeling can guide material selection and interpret interfacial electrostatics in novel solid-state systems [157].

In solid polymer ALMBs, *in operando* and *ex situ* solid-state NMR combined with electron microscopy and surface spectroscopy have been used to quantitatively resolve the relative contributions of SEI formation and dead-lithium accumulation to capacity loss, directly linking the evolution of interfacial chemistry and morphology to coulombic efficiency and guiding electrolyte and interphase design in practical solid-state ALMB configurations [163].

These *in operando* and *postmortem* studies provide a comprehensive understanding of interfacial processes, guiding the rational design of interlayers, surface modifications, and cycling protocols to stabilize alkali-metal deposition across Li, Na, and K anode-less solid-state batteries.

#### 3.2.5. Beyond Li: Na and K Solid-State ALMB Systems

While lithium-based solid-state ALMB have been the primary focus of research, sodium and potassium systems are being increasingly investigated due to their natural abundance and cost advantages. Despite differences in ionic radius, redox potential, and chemical reactivity, many of the interfacial strategies developed for lithium can be adapted to these alternative alkali metals, with specific adjustments to account for their distinct electrochemical behavior.

A pivotal advancement in sodium-based systems is the demonstration of a fully “electrodeless” solid-state architecture, which transcends the conventional anode-less paradigm by eliminating the need for alkali metal plating/stripping while maintaining a defined plateau even at higher current rates. In this configuration, a ferroelectric Na_2.99_Ba_0.005_OCl electrolyte is paired with inert Inconel 625 and aluminum current collectors [157]. The cell operates based on the intrinsic difference in electron chemical potential between the collectors, sustaining a stable discharge plateau (~1.1 V) for months via an interfacial electrostatic double-layer formation. This work not only proves the viability of long-duration Na-based solid-state storage without active anodes but also establishes a new design principle where energy is stored at the interface itself, bypassing many degradation mechanisms tied to metal deposition. Furthermore, it highlights Inconel 625 as an exceptionally stable, corrosion-resistant current collector for highly reactive Na-ion solid electrolytes [157]. It is highlighted that even if the working principle is that of a supercapacitor, the plateau-based discharge is typical of a battery.

Potassium-based solid electrolytes such as K_3_OCl and K_2.99_Ba_0.005_OCl exhibit exceptionally high ionic conductivities (reaching ~120 mS·cm^−1^ at moderate temperatures) along with large ferroelectric dielectric constants, both of which promote enhanced ion mobility and facilitate self-charging behavior in anode-less cells. EIS measurements show that symmetric K-based cells possess low internal resistance and maintain high ionic transport. In contrast, asymmetric configurations employing aluminum or copper current collectors experience significant interfacial resistance, accentuating the need for targeted interface engineering to ensure stable and efficient operation [29].

In sodium ALMB cells, surface modification of copper collectors and the use of microstructured or gradient interlayers have been shown to promote uniform metal deposition, reduce dendritic growth, and enhance cycling stability [77]. These approaches help overcome challenges associated with the larger ionic radius of sodium, which can lead to slower nucleation kinetics and higher susceptibility to void formation. The success of the “electrodeless” sodium cell also highlights the critical importance of current collector chemical stability. *Postmortem* analysis via SEM–EDX and XRD confirmed that the aluminum negative electrode, while mechanically degrading due to residual moisture, did not undergo significant bulk chemical reaction with the ferroelectric electrolyte, whereas the Inconel 625 positive collector remained entirely intact [157]. This maps out a clear path for future work: combining the interfacial electrostatic storage mechanism with optimized, moisture-free assembly to achieve greater longevity and higher specific discharge power [157].

Overall, studies on sodium and potassium solid-state ALMBs illustrate that while the fundamental principles of interlayer and interface engineering are transferable, the design of collector surfaces, interlayers, and cycling protocols must be carefully adapted to the specific electrochemical properties of each alkali metal. These insights expand the toolbox for developing high-performance ALMBs beyond lithium, paving the way for more sustainable and cost-effective energy storage solutions.

Analogously to what was presented in the previous subsection, Table 5 summarizes experimental studies on anode-less and electrodeless cells employing solid electrolytes, organized chronologically from the earliest to the most recent reports.

## 4. Outlook and Perspective: From Lab Bench to Industrial Reality

The encouraging laboratory-scale performance of anode-less batteries highlights their transformative potential for next-generation energy storage technologies. Nevertheless, translating these advances into robust, commercially viable systems demands a systematic and knowledge-driven scaling strategy. This transition must be anchored in a rigorous assessment of the fundamental challenges and degradation mechanisms identified during early-stage research.

Despite the significant progress summarized in this review, three major scientific barriers still limit the practical advancement of anode-less batteries. First, highly reversible metal deposition and stripping under practical current density and areal-capacity conditions remain difficult to achieve, particularly because even small inefficiencies rapidly generate inactive metal and irreversible lithium loss. Second, long-term chemical and mechanical stabilization of the interface remain unresolved, especially in systems where interphase evolution, contact loss, void formation, and resistance growth are strongly coupled. Third, many strategies that appear promising at the material or interface level have not yet been translated convincingly into full-cell architectures operating under practically relevant conditions, including realistic cathode loading, constrained lithium inventory, and manufacturable cell configurations. These three barriers collectively define the key domains that must be systematically refined to transform anode-less batteries from elegant laboratory prototypes into robust and manufacturable technologies.

**1. Mastering the negative electrode interface: for controlled metal deposition**

At the core of anode-less architectures are the reversible, efficient plating and stripping of lithium or sodium onto a bare current collector. Despite its conceptual simplicity, this process presents substantial complexity, centered on three major scientific and engineering challenges.

***(i) Electrolyte engineering***

*How can the electrolyte (particularly in solid-state configurations) be designed to ensure uniform metal nucleation, stabilize interphase layers, and robustly suppress dendritic growth?*

- **Emerging pathway:** The research focus has shifted from seeking pure ionic conductors to developing interface stabilizers. Promising strategies include designing compliant interlayers (e.g., soft polymers, lithiophilic alloys) between the solid electrolyte and current collector and creating hybrid or composite electrolytes that combine ceramic mechanical stability with polymer interfacial wettability. Furthermore, artificial SEI engineering via pretreatment or electrolyte additives aims to create a robust interface from the first cycle.

***(ii) Current collector design***

*In what way can we redesign the surface of the negative current collector?*

- **Emerging pathway:** Passive coatings are evolving into active, multifunctional 3D hosts. These structures are engineered with lithiophilic nucleation sites (e.g., Ag nanoparticles, N-doped carbon) to guide uniform plating, while being sodiophilic or lithiophobic in other regions to confine deposition and prevent top-growth, effectively lowering local current density.

***(iii) Formation protocols***

*What electrochemical conditioning procedures are required during the initial cycles?*

- **Emerging pathway:** The “one-size-fits-all” constant current charge is being replaced by adaptive formation protocols. This involves using low initial currents to build a stable SEI, followed by multistep or pulse plating techniques to refine deposition morphology and maximize initial coulombic efficiency.

Gaining deterministic control over these parameters constitutes the first breakthrough. Yet the anode does not operate in isolation. Its performance is inherently shaped by the rest of the cell, raising the next crucial set of questions.

**2. Configuring the full cell: achieving integrated compatibility and performance**

The anode’s performance is intrinsically linked to the other components. The choices made here determine the final balance between capacity, safety, and cost.

***(iv) Cathode selection***

*What is the ideal cathode material? This choice must reconcile stability, high capacity, and chemical compatibility.*

- **Emerging pathway:** For high energy density, high-nickel layered oxides (NMC) and sulfur cathodes are primary candidates, often requiring protective coatings (e.g., LiNbO_3_) for compatibility with solid electrolytes. For sustainability, manganese-rich layered oxides and iron-based polyanion compounds offer promising, earth-abundant alternatives.

***(v) Defining the operational window***

*Under which conditions of temperature and pressure does the cell reach its optimal balance?*

- **Emerging pathway:** A major focus is reducing the external pressure requirement for solid-state cells by developing mechanically robust, self-standing electrolyte membranes. For temperature, research is directed at wide-temperature-range electrolytes and integrating efficient internal heating elements to ensure operation in subzero conditions.

But overcoming initial inefficiencies is not enough. The true test for any battery technology is its durability over thousands of cycles.

**3. Ensuring longevity and reliability: from cycles to service life**

To achieve this, we must go beyond chemistry and consider intelligent management and monitoring.

***(vi) Electrochemical management***

*How can we implement intelligent charge–discharge protocols?*

- **Emerging pathway:** Advanced battery management system (BMS) algorithms are being researched to incorporate anode potential sensing or impedance tracking. This enables dynamic protocols that adjust based on the cell’s state of health, actively managing the metal inventory and mitigating side reactions over time.

***(vii) System integration and diagnostics***

*What is the role of advanced monitoring?*

- **Emerging pathway:** The development of *in situ* and *operando* techniques (e.g., ultrasonic imaging, pressure cell monitoring) is vital to observe degradation in real time. These data are essential for creating digital twins of the cell, which can predict failure and inform both cell redesign and BMS strategies.

Beyond its role as a conceptual roadmap, the development pyramid also suggests concrete experimental priorities for the field. At the interface level, one key need is for spatially resolved *operando* experiments that directly correlate local stack pressure, contact retention, and void formation with plating–stripping reversibility. At the full-cell level, systematic matrices combining current collector design, electrolyte formulation, and formation protocol should be evaluated under realistic cathode loading and constrained lithium inventory, rather than in isolated half-cell or low-loading conditions. For long-term reliability, a particularly important direction is the combined use of electrochemical testing with inactive-lithium quantification and *operando* diagnostics to distinguish SEI growth, dead-metal accumulation, and contact loss-driven degradation. Framed in this way, the pyramid is intended not only to organize existing knowledge, but also to identify experimentally testable and translation-relevant research priorities.

The development pyramid should therefore be understood as a qualitative translation framework rather than a strict TRL classification. Its lower layer, focused on mastering the negative electrode interface, corresponds primarily to early-stage validation of interfacial reversibility and controlled metal deposition, often demonstrated in simplified cells or small format full cells. The middle layer, focused on full-cell configuration, reflects intermediate maturity, where promising interface and electrolyte strategies must remain effective under realistic cathode loading, constrained lithium inventory, and more integrated architectures. The upper layer, focused on longevity and reliability, corresponds to the stage at which long-term stability, diagnostics, control strategies, and manufacturability become decisive, including translation toward pouch-cell formats and industrially relevant operating conditions. In this sense, the industrial examples discussed below should be interpreted as practical reference points along this progression, rather than as a strict validation of the pyramid through fixed TRL numbers.

Reported energy-density values in anode-less batteries should be interpreted with caution unless the level of calculation is clearly specified. Theoretical or material-level estimates, laboratory cell-level values, projected pouch-cell metrics, and practical stack-level energy densities are not directly equivalent. Stack-level values may be substantially reduced by inactive components such as packaging, excess electrolytes, current collectors, separator or solid-electrolyte thickness, tabs, and in some solid-state configurations pressure-maintenance hardware. Therefore, very high reported energy-density values should not be considered directly representative of practical stack-level performance unless the underlying assumptions and cell architecture are explicitly stated.

These emerging strategies are not confined to scientific articles. On the contrary, they are being rapidly adopted and tested within a vibrant industrial landscape, where the anode-less concept is undergoing the decisive test of scalability and commercial viability. The journey towards manufacturing reality is already underway, driven by a dynamic ecosystem of startups, industrial giants, and strategic partnerships working to transform the anode-less concept into viable products. This collective effort is materializing along two parallel technological pathways.

**1. The all-solid-state pathway (high-potential future)**

Companies such as QuantumScape, Solid Power, and SES AI are leading the development of all-solid-state cells with anode-less architecture. They are currently in the critical phase of prototype validation and operating dedicated pilot production lines. The focus is on solving the final challenges of material and process scalability, with support and funding from automotive consortia (e.g., Volkswagen, Ford, BMW). Their market entry window is projected for 2027–2030, although this timeline still depends on overcoming key scale-up, interfacial, and manufacturing constraints. Despite this momentum, major challenges remain for the all-solid-state industrial pathway. Public disclosures and recent technical literature indicate that key unresolved issues include the scalable production of thin and defect-tolerant solid electrolyte separators, the maintenance of uniform interfacial contact over large cell areas, the control of pressure inhomogeneity and cell expansion during cycling, and the integration of these materials into manufacturing routes compatible with automotive cost and throughput targets. In addition, even when promising electrochemical performance is demonstrated at prototype scale, translation to large-format cells remains constrained by process yield, stack-level mechanical design, and the need to preserve performance under practically relevant pressure conditions.

**2. The hybrid pathway and the first commercial reality (present)**

CATL, the world’s largest battery manufacturer, has turned the anode-less principle into a commercial reality through a pragmatic approach. Its “condensed battery” utilizes a semisolid electrolyte that enables the use of a bare current collector, with lithium forming *in situ* during the first charge. This technology, with reported energy densities exceeding 500 Wh·kg^−1^ (at the cell level), has already entered production and has been selected for niche applications such as electric aviation. Nevertheless, these values should be interpreted with caution, since practical stack-level energy density may be significantly lower once packaging and other inactive components are included. CATL thus illustrates that the anode-less design principle can become commercially relevant when embedded within a broader hybrid material platform, thereby accelerating industrial interest in this class of architecture. At the same time, the condensed-battery example should be interpreted with caution as a validation of a specific hybrid material platform rather than as a direct validation of the anode-less concept in isolation. Because the detailed electrolyte formulation, lithium inventory management strategy, and cathode design remain proprietary, it is not yet possible to quantify how much of the reported cell-level energy density derives specifically from the anode-less architecture, as opposed to the broader contribution of semisolid electrolyte chemistry, cell engineering, and high-energy cathode integration. For this reason, this case is best viewed as evidence that anode-less design can become commercially relevant within a broader hybrid material package, rather than as a clean benchmark of architecture-only benefit.

This dual pathway creates a phased and robust industrial roadmap: a successful short-term hybrid implementation (CATL) can help fund and validate the race towards the next generation of pure solid-state technology led by startups and industrial consortia. The bridge between the laboratory and the factory is not only built, but has already begun to be crossed.

## Figures and Tables

**Table 1:** Comparison of representative recent overview articles on anode-less batteries and the scope of the present work.

Ref.	Focus	System/Chemistry Scope	Key Emphasis	How the Present Review Differs from Cited
[18]	General review of anode-less lithium batteries	Li anode-less batteries	Challenges, strategies, failure mechanisms, and characterization	Broader fundamentals-to-application framework
[19]	Critical analysis of anode-less cell concepts and practical battery development	Anode-less batteries in liquid and solid electrolyte systems, including Li and Na plating contexts	Practical cell performance, plating/stripping mechanisms at the negative current collector, degradation processes, influence of other cell components, and gaps in data accessibility, reporting standards, and metrics	Broader fundamentals to future directions framework with stronger emphasis on conceptual organization and development roadmap
[16]	Review of post-lithium anode-less metal batteries	Post-lithium systems, including Na, K, and Zn	Challenges, reversibility, and strategies beyond Li	Architecture-centered rather than chemistry-centered
[15]	Interface engineering in anode-less sodium batteries	Sodium anode-less batteries	Interfacial chemistry, Na deposition/stripping, and stabilization strategies	Broader than interface-focused sodium scope
[20]	Broad overview of anode-less batteries	Broad anode-less battery landscape	Mechanisms, challenges, opportunities, energy density, and safety	Structured around a conceptual pyramid and development roadmap
[21]	SEI-focused review with characterization emphasis	Lithium-metal and anode-less lithium batteries	SEI formation, composition, and *in situ*/*operando* characterization	Broader than SEI characterization alone
[22]	Mechanistic review of active lithium loss in anode-less batteries	Li anode-less lithium-metal batteries	Active lithium loss, degradation mechanisms, and mitigation strategies	Broader than single-mechanism degradation analysis
[4]	Focused review on sulfide-based of anode-less batteries	Sulfide-based anode-less solid-state batteries	Sulfide interfaces, non-uniform Li growth, voids, and solid-state solutions	Broader than sulfide-based solid-state scope

**Table 2:** Summary of key characterization techniques for anode-less batteries, their objectives, and representative references.

Category	Technique	Main Information Provided	Mechanistic Relevance	Example Applications	References
**Electrochemical analyses**	Charge–discharge cycling 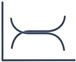	Capacity, coulombic efficiency, reversibility, stability	Overall cycling instability, active lithium loss, and reversibility limitations	Distinguishes stable Li nucleation vs. interfacial degradation; validates separators and frameworks; conditioning effects	[26,39,40,55,56,57,58,59,60,61,62,63,64]
	PEIS 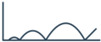	Internal resistance, charge transfer, interfacial properties	Interfacial degradation, contact loss, and resistance buildup during cycling	Nucleation engineering; polymer separator integration; *in situ* polymerization; in-series solid-state cells	[26,38,65]
	CV 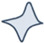	Redox processes, interfacial reactions, stability	Nucleation behavior, plating/stripping reversibility, and reaction kinetics	Preconditioning; polymerization peaks; redox stability of SSEs; ferroelectric systems	[26,38,65,66]
	GITT 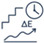	Ionic diffusivity, kinetic limitations	Diffusion/transport limitations and polarization-related kinetic bottlenecks	Diffusion coefficients in elastic networks; dual-seed strategies; Ah-level separators; Zn^2+^ transport stabilization	[39,40,67,68]
**Interfacial potential mapping**	SKP 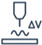	Macroscopic surface potential/work function mapping	Surface chemical-potential gradients and interfacial stability evolution	Surface potentials shift in heterocells; dielectric oxides vs. SSEs; Li alloying/plating in 3D collectors	[70,71,72]
	KPFM 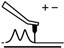	Nanoscale CPD mapping	Local interfacial heterogeneity, nucleation-active regions, and surface instability	Li nucleation pathways; *in operando* redistribution; suppressed gradients; dendrite nucleation at grain boundaries; combined with AFM, ToF-SIMS	[73,74,75,76]
**Structural and chemical analyses**	XRD 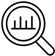	Crystal structure, phase transitions	Structural transformation pathways and reaction-product formation	Cathode lattice variations; Li–Ag alloy phases; reversible alloy phases; lithiophilic interlayers	[40,48,78,79]
	XPS 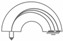	Surface chemistry, SEI/interphase composition	Interphase formation, surface decomposition pathways, and chemical instability	Wetting layers on Cu; SEI composition (organic/inorganic); conditioning effects; high-entropy SEIs (Na); degradation pathways	[21,26,80,81,82]
	ToF-SIMS 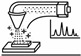	Nanoscale chemical mapping	Interphase nonuniformity and depth-dependent chemical degradation	Lithiophilic seeds, dual-metal interlayers, near-surface Li-ion irrigation; lithio-amphiphilic bilayers; Na/K SEIs	[55,57,58,83,84,85,86,87]
	TEM 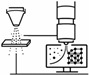	Interfacial structures, deposition morphology, phase transformations	Nanoscale interfacial degradation, filament formation, and local structural failure	Metallic interlayers; Sn-decorated fibers; electron-deficient collectors; engineered coatings; conversion-type anodes; Na collectors; Cu substrates	[48,50,88,89,90,91,92,93,94]
	LIBS 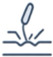	Surface/interfacial elemental analysis, depth profiling	Spatially heterogeneous degradation and nonuniform elemental redistribution	Interfacial chemistry in SSEs; cycling-induced compositional changes; *postmortem* analysis; Li detection	[26,31,95,96]
	*In operando* techniques (XRD, TEM, XPS, Raman) 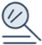	Real-time structural/chemical/morphological changes	Real-time interfacial evolution, reaction pathways, and failure development during cycling	Phase transitions; Li nucleation and dendrites; SEI chemistry; polydopamine coatings; Li–S polysulfides; Na SEI; oriented Cu	[50,81,88,94,97,98,99,100]
**Morphological studies**	SEM–EDX 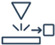	Surface morphology, elemental distribution	Surface degradation, deposit morphology, and compositional heterogeneity	Li plating/stripping (not detected directly), interphase degradation, Ag distribution, conditioning cells, Na deposition, surface modifications	[26,101,102,103,104,105,106]
	AFM 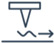	Nanoscale morphology, nucleation sites	Surface roughness evolution, local mechanical heterogeneity, and early deposition instability	ZnF_2_ coatings; Li–S systems; *in operando* Zn nucleation; engineered collectors and electrolytes	[41,68,100,107,108,109,110,111]
	*In operando* tomography 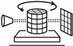	3D real-time morphological evolution	Void formation, filament growth, and 3D morphological evolution during cycling	Zero-excess Li failure suppression; machine learning-enhanced tomography; multiscale tomography/diffraction coupling	[113,114,115,116,117,118]
**Thermal analyses**	*In operando* calorimetry 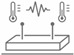	Heat generation during cycling, parasitic reactions	Heat generation pathways, parasitic reactions, and thermal signatures of interfacial instability during cycling	Failure diagnosis in Li–S (potential for anode-less systems)	[119]
	DSC 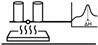	Thermal stability, phase transitions	Thermal reactivity, decomposition behavior, and safety-related phase or interfacial instability	Seawater Na batteries; Zn co-solvent electrolytes; deep eutectic solvents; glass-derived SSEs	[103,120,121,122]
	ARC 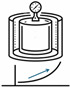	Thermal runaway and abuse testing	Thermal runaway propensity, self-heating behavior, and abuse-response safety limits	Safety margins in Li-metal SSEs; ultralight integrated anodes; porous carbons for heat dissipation	[123,124]

**Table 3:** Summary of experimental studies on anode-less cells employing liquid electrolytes, organized chronologically from the earliest to the most recent reports.

Year	Cell Type	Cell Configuration	Electrolyte	Interlayer/Surface Modification	Key Results/Performance	Ref.
2016	Coin	Cu||NMC_622_	LiPF_6_ in EC/DMC	Cu current collector, focus on fundamentals of anode-less Li metal plating	>100 cycles, CE > 95%, stable Li plating/stripping with minimal dendrite formation	[23]
2017	Coin	Cu||carbon coated NVP composite	NaPF_6_ in EC/DE	*In situ* plating of sodium metal on a bare Cu current collector	>150 cycles, CE > 95%, stable Na plating/stripping, minimized dead sodium	[144]
2019	Coin	Cu||NMC_811_	Concentrated dual salt electrolyte: LiFSI and LiPF_6_ in EC/EMC	Electrolyte induced stable SEI formation on lithium metal	>250 cycles, CE > 97%, improved cycling stability and lithium-metal protection	[136]
2019	Pouch	Cu||NMC_532_	Dual-salt liquid electrolyte: LiPF_6_ and LiTFSI in EC/EMC	Formation of stable SEI promoted by dual-salt electrolyte	>300 cycles, CE > 98%, dendrite-free lithium morphology, enhanced cycling stability	[135]
2020	Coin	Cu||NMC_532_	Optimized electrolyte: baseline is a conventional carbonate	Morphological control via electrolyte tuning to mitigate failure mechanisms	>200 cycles, CE > 96%, reduced dendrite formation, improved cycle life	[143]
2020	Coin	Cu||LFP	LiFSI in DME/BTFE	Electrolyte chemistry optimizing SEI formation and lithium plating behavior	>150 cycles, CE > 95%, improved stability without lithium-metal anode	[133]
2021	Coin	Cu||LFP	LiFSI in P_1114_FSI ionic liquid	Electrolyte enabled stable SEI and improved lithium plating	>200 cycles, CE > 98%, enhanced cycling stability and dendrite suppression	[139]
2021	Coin	Cu||NMC_622_	LiFSI, LiTFSI and LiNO_3_ in DME/DOL	Electrolyte induced uniform lithium deposition and stable SEI formation	>300 cycles, CE > 99%, enhanced cycling stability, dendrite suppression	[137]
2021	Coin	Cu||NMC_811_	LiPF_6_ in EC/EMC/Triglyme	None or intrinsic interface formation promoted by electrolyte design	>200 cycles, CE > 98%, improved stability and dendrite suppression	[134]
2021	Pouch	Cu||NMC_811_	65 different electrolyte formulations: based on carbonate solvents (EC: EMC) with varied salts (LiPF_6_) and a wide range of additives	None or electrolyte-driven SEI modifications studied	>300 cycles, CE > 99%, electrolyte dependent cycling stability	[138]
2022	Coin	Cu||NMC_811_	LiFSI in a mixture of DME and TTE	Interface engineering for homogeneous lithium plating	>300 cycles, CE > 99%, uniform Li morphology, suppressed dendrites	[149]
2022	Coin	3D Cu-based hierarchical host||NVP	NaPF_6_ in EC: DEC	3D hierarchical sodiophilic host structure to promote uniform Na plating	>300 cycles, CE > 99%, enhanced Na utilization, suppressed dendrites and dead sodium formation	[150]
2022	Coin	Cu||NaMnO_2_	Beyond concentrated sodium salt electrolyte tailored for high voltage	Electrolyte engineered interface for stable sodium plating	80% capacity retention after 350 cycles, CE > 97%, high voltage stability, suppressed dendrites	[140]
2023	Coin	Cu||NVP	NaPF_6_ in EC: DEC with FEC	HCOONa artificial layer formed by HCOOH vapor pretreatment	80% capacity retention after 350 cycles, average CE 99.69%, stable Na plating, suppressed dendrites	[127]
2023	Coin	Cu||NVP	NaPF_6_ in EC: PC: EMC with FEC	*In situ* formed Sn-Cu alloy layer from SnO_2_ coating	80% capacity retention after 400 cycles, average CE ~99.5%	[126]
2023	Coin and pouch	Cu||KPTCDA	KPF_6_ in DME and PDMS	*In situ* formed interfacial layer via polydimethylsiloxane (PDMS) additive, creating a potassiophilic interface and robust organic-inorganic hybrid SEI	99.80% average CE at −40 °C, 82% capacity retention after 50 cycles	[148]
2024	Coin	Cu||LFP	LiTFSI in DOL/DME and LiNO_3_	No surface modification or interlayer was applied	Achieves the lowest dead lithium content (2.7 µAh), a high average Coulombic efficiency of ~96%, and the longest cycle life	[147]
2024	Coin	Cu||NVP	NaPF_6_ in EC: DEC with FEC	Semi-coherent Cu_2_Sb alloy interface	80.5% capacity retention after 500 cycles, average CE ~99.8%	[125]
2024	Coin	Cu||LFP	Entropy-driven 60 mol% LiF-LiI halide solid electrolyte	Electrolyte—driven interface enabling stable lithium plating without metal anode	Stable cycling for >400 cycles, average CE ~99.4%	[141]
2024	Pouch	MgF_2_@NCHNFs||NVP	NaTFSI in FEC:P	3D host of nitrogen-doped carbon hollow nanofibers with MgF_2_ that, during initial plating, forms an *in situ* gradient fluorinated alloy architecture with a surface NaF layer and subsurface Mg site	Dendrite free Na deposition at high rates (10 mA·cm^−2^) and capacities (10 mAh.cm^−2^), achieving > 500 h stability in symmetric cells and 96% capacity retention after 400 cycles in pouch cells	[131]
2024	Pouch	Cu||NMC_811_	LiPF_6_ in EC/DEC; dual-salt electrolyte (DSE) and localized high-concentration electrolyte (LHCE)	None: the study focused on optimizing the formation protocol (initial charging rate), with no current collector surface modification	~20% capacity retention after 20 cycles, optimal formation at max non-dendritic rate (C/2 for standard electrolyte)	[146]
2024	Coin	Cu||NVP	NaPF_6_ in EC: PC with FEC	High-entropy SEI formed by edge electron effect to enhance durability on N-doped carbon nanotube modified Cu	86.5% capacity retention after 500 cycles, average CE ~99.6%	[81]
2025	Coin	Al||PTPAn	KPF_6_/KTFSI-DME/diglyme	Anion derived SEI formed *in situ* via KTFSI salt additive, creating a robust, inorganic-rich interface	99.98% average CE at −40 °C, 407 Wh·kg^−1^ energy density, 80% capacity retention after 183 cycles	[142]
2025	Coin	Cu||LFP	LiFSI in DME	Holey graphene (HG) or graphene (GR) coated as an interlayer on the Cu	HG-based achieved 75% capacity retention after 200 cycles, CE > 99.6%	[129]
2025	Coin	Cu||NMC_811_	LiPF_6_ in EC: EMC with VC	Lithium carbide prelithiation agent to improve initial lithium inventory and cycling stability	80% capacity retention after 420 cycles, CE > 99.1%	[145]
2025	Coin	Cu||LFP	LiPF_6_ in EC: DEC with FEC	Protonated polyaniline coating on Cu to improve lithium nucleation	Stable cycling for 200 cycles, average CE ~99.2%, uniform Li deposition	[111]
2025	Coin	Cu||NMC_111_	LiPF_6_ in EC: EMC with LiDFOB sacrificial salt	Sacrificial lithium salts favoring stable SEI and CEI formation	Feasibility analysis for anode-less cells, CE ~99.1% in Cu	[47]
2025	Coin	SnSb/C-Cu foam||NVP	NaPF_6_ in EC: PC with FEC	3D sodiophilic SnSb/C-coated Cu foam host	85% DOD reversibility, stable for 400 cycles, average CE ~99.5%, dendrite suppression	[130]
2025	Coin	PBA@Cu||NVP PBA@Cu||LFP	NaPF_6_ in G2; LiTFSI in DME/DOL and LiNO_3_	*In situ* integrated Prussian blue analogue (PBA) interlayer on Cu	75.3% capacity retention after 300 cycles at 5C and an average CE of ~99.7%	[132]
2025	Coin	Cu_2_O on Cu||LFP	LiPF_6_ in EC: EMC with FEC	3D Cu foam current collector with lithiophilic Cu_2_O coating	92.8% capacity retention after 200 cycles, average CE ~99.2%	[42]

**Table 4:** Comparative features of liquid- and solid-state anode-less battery systems.

Aspect	Liquid-Electrolyte Anode-Less Systems	Solid-State Anode-Less Systems	Main Implication
Interfacial contact	Assisted by electrolyte wetting and dynamic interfacial accommodation	Limited by solid–solid contact, surface conformity, and contact retention during cycling	Solid-state systems are more sensitive to local interfacial defects and contact loss
Current-density tolerance	Can often be improved through electrolyte formulation, additives, and current collector design, but remains sensitive to nonuniform plating and parasitic reactions	Frequently constrained by local current constriction, interfacial resistance, and chemomechanical instability	Stable high-rate operation is generally more challenging in solid-state systems
Pressure requirements	Usually low to moderate	Often significant to maintain interfacial contact and suppress void formation during stripping/plating	Pressure is a major design variable in solid state
Interfacial resistance evolution	Strongly influenced by SEI composition, electrolyte depletion, and continuous side reactions	Strongly influenced by contact loss, void formation, and interphase growth at solid–solid interfaces	The dominant origins of resistance growth differ substantially between the two systems
Practical energy-density considerations	Potentially high, but strongly affected by electrolyte excess, inactive components, and lithium inventory loss	High theoretical promise, but stack pressure hardware, SSE thickness, and packaging constraints may reduce practical gains	Stack-level metrics must be critically interpreted in both cases

**Table 5:** Summary of experimental studies on anode-less cells employing solid electrolytes, organized chronologically from the earliest to the most recent reports.

Year	Cell Type	Cell Configuration	Electrolyte	Interlayer/Surface Modification	Key Results/Performance	Ref.
2016	Symmetric (hermetically sealed in epoxy resin)	Cu||Cu Li||Li	Li_2.99_Ba_0.005_OCl_1-x_(OH)_x_ and Na_2.99_Ba_0.005_OCl_1-x_(OH)_x_	No surface modification or interlayer reported; the glassy electrolyte is applied directly onto the copper or lithium current collector	Demonstrated high ionic conductivity (~best liquid), low activation energy (~0.06 eV), stable dendrite-free Li/Na plating/stripping for 19 days, and anomalous self-charging behavior	[24]
2000	Thin film	Cu||LiCoO_2_	LiPON	A dense overlayer (LiPON or Parylene C) was deposited on the copper current collector; this layer was crucial to confine the plated lithium and prevent detrimental side reactions, enabling stable long-term cycling	Achieved > 1000 cycles via *in situ*-plated Li anode enabled by a protective overlayer	[156]
2003	Thin film	SS||Li_x_V_2_O_5_	LiPON	No interlayer: lithium anode formed *in situ* during first charge between SS and LiPON	Li plating during first charge; stable cycling over 100 cycles; discharge capacity of 110 mAh g^−1^ at 5 μA cm^−2^	[168]
2019	Thin film	LiPON–Cu–LiPON||LCO/Al_2_O_3_	LiPON	Artificial LiPON–LiPON interface created by depositing a second LiPON layer over Cu current collector	Li metal deposits and propagates along the artificial LiPON–LiPON interface but does not penetrate through bulk LiPON, confirming its ability to suppress Li dendrites; planar Li growth observed over ~2 mm distance	[167]
2020	Custom research	Cu||PEO-NCA	LLZO	No interlayer or surface modification reported for the anode interface; the cathode interface uses PEO–LiTFSI as a contact/composite layer	Demonstrated “Li-free” solid-state battery *in situ* plated Li anodes (up to 5 mAh.cm^−2^) achieving ~100% CE over 50 cycles without LLZO degradation	[176]
2020	Coin	Cu||NMC_111_	LLZTO–PEO composite solid electrolyte (PEO–LiTFSI with 10 wt% LLZTO filler)	Ultrathin (7–10 μm) LLZTO–PEO–CPE layer spin-coated on both Cu current collector and NMC cathode surfaces	Achieved high ionic conductivity, 4.76 × 10^−4^ S·cm^−1^ at RT; dendrite free Li, and 98.8% average CE with 41.2% capacity retention after 65 cycles at 0.2 mA·cm^−2^	[165]
2021	Coin	Cu||LTO	LPSCI and LGPS	A Mo-sputtered current collector and an LPSCI interlayer were used for *operando* analysis and to isolate interface effects, with no surface modification applied to the copper	LPSCI electrolyte achieved efficient lithium plating with stable SEI formation after initial decomposition, while LGPS showed continuous electrolyte degradation without lithium deposition due to conductive SEI formation	[162]
2022	Coin	Cu||NMC_622_	LPSCl	Unmodified Cu and carbon-coated Cu	<50 cycles, low CE, heterogeneous Li plating and void formation quantified as root cause of rapid capacity fade	[158]
2022	Pouch	Cu with Ag-C||LFP	PEO–LiTFSI with Al_2_O_3_ nanoparticles	Ag–C nanocomposite coating on Cu foil	Doubled initial discharge capacity (93 vs. 46 mAh·g^−1^) vs. bare Cu; CE > 99% after 50 cycles; 54% capacity retention after 50 cycles	[104]
2023	Pouch	Cu and Cu with ZnO||LFP	Hybrid electrolyte: Li_2.99_Ba_0.005_ClO glass ceramic ferroelectric matrix impregnated in cellulose with PVA_c_ binder	ZnO interlayer used to promote uniform lithium nucleation; electrochemical conditioning with controlled load resistors to optimize interface and cycling stability	Confirmed uniform lithium plating (~2.9 µm thick); cells with ZnO layer deliver higher discharge power and longer retention; cycling efficiency over 99%; stable discharge plateau ~3.2 V indicating high lithium chemical potential	[26]
2023	Coin	Cu||LFP	PEO, PTMC, and PCL with LiTFSI	No surface modification or interlayer	Demonstrated the feasibility of anode-less cells with solid polymer electrolyte; CE limited by “dead lithium” formation; need to improve lithium deposition morphology	[173]
2024	Custom-pressurized	SS||Li	LPSCl	No surface modification or interlayer was mentioned; the current collector is plain SS	High temperature (80 °C) promoted uniform, film like Li morphology with 100% active area and dominant horizontal growth, whereas low temperature (25 °C) led to vertical growth, discontinuous islands, and reduced active area (63%), with the study focusing solely on the first deposition	[160]
2024	Coin	Ag–C buffer layer||NMC811	LPSCl	Ag–C buffer layer facilitating lithiation/delithiation and enhancing interfacial stability	>250 cycles, CE > 96%, improved interface stability and lithium plating/stripping efficiency	[175]
2024	Coin	Cu||LFP	PDOL polymer electrolyte with LiPF_6_–LiFSI–LiFSI and CsClO_4_	SEI modification via electrolyte composition	LiFSI + CsClO_4_ electrolyte achieved 89.4% initial efficiency and 98.5% stable efficiency by reducing dead lithium formation by 20× compared to conventional electrolyte	[163]
2024	Pouch	Cu with ZnO and Cu with Li_2_O||LFP	Hybrid electrolyte: Li_2.99_Ba_0.005_OCl glass ceramic ferroelectric matrix impregnated in cellulose with PVAc binder	ZnO or Li_2_O nucleation layer was doctor bladed onto the copper current collector to enhance lithium-metal plating	Single ZnO-based cells achieved a 76% CE and 0.82 mAh capacity, while series-connected cells powered LEDs for multiple cycles with a final voltage of 5.3 V	[38]
2024	Coin	Cu||NMC811	*In situ* polymerized electrolyte with liquid metal additives (LM-PE)	Liquid metal (Ga–In–Sn alloy) nanoparticles as nucleation seeds on Cu current collector	Achieved 80% capacity retention after 150 cycles; high average CE of 99.2%; stable Li plating/stripping with dense; dendrite-free morphology	[152]
2024	Coin	Ag–In coated SUS||NMC811	Li_3_PSeCl_2_Br_0.5_	In-doped Ag metal coating on SUS current collector via CCS sputtering	80% capacity retention after 250 cycles at 1C with 99.8% CE, enabled by stable Li–Ag–In alloy interlayer	[170]
2024	Coin	Cu/Ag–C||NMC	LPSCl	Ag–C nanocomposite interlayer (Ag nanoparticles and carbon black) coated on Cu	Optimal assembly pressure (500–530 MPa) achieved 198.1 mAh·g^−1^ initial capacity and 410 Wh·kg^−1^ energy density by enhancing interfacial adhesion, while higher pressures caused electrolyte fracture and rapid performance decay	[159]
2024	Coin	Cu||NMC811	Polyester-based polymer electrolyte	Investigation of lithium loss mechanisms at the polymer electrolyte–anode interface	>200 cycles, CE > 95%, lithium loss quantified impacting cycling stability and interface durability	[172]
2024	Pouch	Cu||Zn	Hybrid electrolyte: Na_2.99_Ba_0.005_OCl glass ceramic ferroelectric matrix impregnated in cellulose with PVAc binder	The copper and zinc current collectors were sanded to create texture for improved sodium nucleation; a carbon felt interlayer was added adjacent to the positive copper current collector	Electrodeless sodium pouch cells demonstrated self-charging, with voltage increasing during discharge to 1.2 V and confirmed sodium metal plating on zinc	[66]
2025	Custom pressurized	SS||Li	LPSCl	No surface modification or interlayer was used; the current collector was plain SS	Low stack pressure (2 MPa) caused early failure due to irregular plating and Li filaments, while high pressure (20 MPa) induced electrolyte fracture, with optimal performance at 10 MPa achieving 4.56 mAh·cm^−2^	[161]
2025	Pouch	Ag–C||NCA	LPSCl	Ag–C nanocomposite interlayer	20 MPa pressure during storage reduces cathode degradation at high SOC, improving capacity retention to ~89% over 200 cycles	[171]
2025	Coin	Cu||Li	Nb–LLZO	Cu current collector (50–1200 nm) was deposited by sputtering directly onto polished LLZO (~100 nm roughness), without any additional interlayers or lithiophilic materials	Cells operated for ~180 h (40 cycles) without external pressure, with 75–90% CE, until short circuit due to dendrites; Li morphology varied with Cu thickness, with preferential nucleation along polishing lines and interfacial resistance reduction from 3187 Ω to 82 Ω	[112]
2025	Pouch	Microstructured Cu||Zn	Hybrid electrolyte: Na_2.99_Ba_0.005_OCl glass ceramic ferroelectric matrix impregnated in cellulose with PVAc binder	The positive copper current collector was microstructured using colloidal lithography and O_2_ plasma reactive ion etching (0–15 min) to create a hexagonal pattern of micro-cones; this process increased the surface area by up to ~30% and modified the surface chemical potential	Cell performance was optimized with a 12-min etched copper collector, where its surface properties proved more critical than its increased area for boosting conductivity and capacity	[77]
2025	Coin	SS|MoS_2_||LiNbO_3_-coated NMC622	LPSCl	MoS_2_ thin film on current collector forms artificial SEI (Li_2_S + Mo)	MoS_2_—15 m cell achieved 161.1 mAh·g^−1^, 58.9% retention (20 cycles), and 96.7% CE	[177]
2025	Coin	Stainless steel|Ag–C interlayer||NMC811	LPSCl	Spray-printed Ag–C nanocomposite bilayer (Ag-rich at current collector and CB-rich at electrolyte)	Structured Ag–C bilayer improved initial discharge capacity to >190 mAh·g^−1^ and capacity retention vs. unstructured interlayer; >98% CE over 100 cycles; promoted uniform Li plating directly on CC	[101]
2025	Coin	Cu–Ag interlayer||NMC111	LLZO	Ag interlayer (200 nm) on LLZO forms Li–Ag alloy for uniform Li plating	~86% CE, >960 cycles in half-cell, operates up to 0.9 mA·cm^−2^ in full-cell, dendrite-free Li plating via Li–Ag alloy mechanism	[88]
2025	Coin	Cu||NMC811	LPSCl	Lithiophilic Au–Sn dual-metal layer on the Cu	>400 cycles, CE > 99.5%, kinetically controlled Li plating via Au–Sn layer enabling uniform deposition and superior cyclability	[58]
2025	Coin	Cu||NMC811	LPSCl	*In situ* silver exsolution from electrolyte at Cu interface	~99.7% average CE, 100+ cycles stability at 1.2 mAh·cm^−2^ via *in situ* Ag interlayer in all-solid-state cell	[169]
2025	Pouch	Cu/ZnO + C||LFP	Li_2.99_Ba_0.005_OCl	ZnO and active carbon coating on Cu	Stable Li plating confirmed via LIBS mapping, achieving 0.16 µm Li thickness and 2.10 V discharge plateau over 219 h in anode-less cells	[31]
2025	Coin	Cu||LFP	Solid glassy electrolyte Li_3_PS_4_–P_2_O_5_ composite film	Solid glassy electrolyte film on current collector to enable dendrite-free lithium plating	>200 cycles, CE > 97%, dendrite suppression, enhanced interfacial stability	[164]
2025	Coin	Ti||LCO	LiPON	Unmodified Ti surface	Higher current densities (2.5 mA·cm^−2^) enable uniform Li deposition with dense columnar morphology and reduce interfacial resistance	[166]

## Data Availability

Data sharing is not applicable.

## Abbreviations

The following abbreviations are used in this manuscript.

AFM	atomic force microscopy
ALBs	anode-less batteries
ALMBs	anode-less lithium-metal batteries
ALSSBs	anode-less solid-state batteries
AM	additive manufacturing
ARC	accelerating rate calorimetry
BMS	battery management system
CC	current collector
CE	coulombic efficiency
CPD	contact potential difference
CV	cyclic voltammetry
DFAM	design for additive manufacturing
DFT	density functional theory
DMC	dimethyl carbonate
DME	dimethoxyethane
DSC	differential scanning calorimetry
EC	ethylene carbonate
EDX	energy-dispersive X-ray spectroscopy
FFF	fused filament fabrication
GITT	galvanostatic intermittent titration technique
HG	holey graphene
KPFM	Kelvin probe force microscopy
LFP	lithium iron phosphate (LiFePO_4_)
LIBS	laser-induced breakdown spectroscopy
LIPON	lithium phosphorus oxynitride
LLZO	lithium lanthanum zirconium oxide (Li_7_La_3_Zr_2_O_12_)
LPSCl	lithium phosphorus sulfur chloride (Li_6_PS_5_Cl)
MBs	metal batteries
ML	machine learning
NMC111	nickel manganese cobalt oxide (LiNi_0.33_Mn_0.33_Co_0.33_O_2_)
NMC532	nickel manganese cobalt oxide (LiNi_0.5_Mn_0.3_Co_0.2_O_2_)
NMC622	nickel manganese cobalt oxide (LiNi_0.6_Mn_0.2_Co_0.2_O_2_)
NMC811	nickel manganese cobalt oxide (LiNi_0.8_Mn_0.1_Co_0.1_O_2_)
PBAs	Prussian blue analogues
PDMS	polydimethylsiloxane
PEIS	potentiostatic electrochemical impedance spectroscopy
PEO	polyethylene oxide
PVDF	polyvinylidene fluoride
SEI	solid-electrolyte interphase
SEM	scanning electron microscopy
SHE	standard hydrogen electrode
SKP	scanning Kelvin probe
SS	stainless steel
SSE	solid-state electrolyte
SUS	sustained discharge
TEM	transmission electron microscopy
TMSP	trimethylsilyl phosphate
ToF-SIMS	time-of-flight secondary ion mass spectrometry
XPS	X-ray photoelectron spectroscopy
XRD	X-ray diffraction
